# Improved diagnosis of thyroid cancer aided with deep learning applied to sonographic text reports: a retrospective, multi-cohort, diagnostic study

**DOI:** 10.20892/j.issn.2095-3941.2020.0509

**Published:** 2021-09-07

**Authors:** Qiang Zhang, Sheng Zhang, Jianxin Li, Yi Pan, Jing Zhao, Yixing Feng, Yanhui Zhao, Xiaoqing Wang, Zhiming Zheng, Xiangming Yang, Lixia Liu, Chunxin Qin, Ke Zhao, Xiaonan Liu, Caixia Li, Liuyang Zhang, Chunrui Yang, Na Zhuo, Hong Zhang, Jie Liu, Jinglei Gao, Xiaoling Di, Fanbo Meng, Wei Ji, Meng Yang, Xiaojie Xin, Xi Wei, Rui Jin, Lun Zhang, Xudong Wang, Fengju Song, Xiangqian Zheng, Ming Gao, Kexin Chen, Xiangchun Li

**Affiliations:** 1 Department of Maxillofacial and Otorhinolaryngology Oncology, National Clinical Research Center for Cancer, Key Laboratory of Molecular Cancer Epidemiology of Tianjin, Key Laboratory of Cancer Prevention and Therapy of Tianjin, Tianjin Medical University Cancer Institute and Hospital, Tianjin Medical University, Tianjin 300060, China; 2 Department of Diagnostic and Therapeutic Ultrasonography, National Clinical Research Center for Cancer, Key Laboratory of Molecular Cancer Epidemiology of Tianjin, Key Laboratory of Cancer Prevention and Therapy of Tianjin, Tianjin Medical University Cancer Institute and Hospital, Tianjin Medical University, Tianjin 300060, China; 3 Department of Ultrasonography, Weihai Municipal Hospital, Cheeloo College of Medicine, Shandong University, Weihai 262400, China; 4 Department of Pathology, National Clinical Research Center for Cancer, Key Laboratory of Molecular Cancer Epidemiology of Tianjin, Key Laboratory of Cancer Prevention and Therapy of Tianjin, Tianjin Medical University Cancer Institute and Hospital, Tianjin Medical University, Tianjin 300060, China; 5 Department of Ultrasonography, Affiliated Hospital of Chifeng University, Chifeng 024000, China; 6 Department of Ultrasonography, Integrated Traditional Chinese and Western Medicine Hospital of Jilin city Jilin Province, Jilin 132000, China; 7 Department of Ultrasonography, Dezhou Municipal Hospital, Dezhou 253000, China; 8 Department of Ultrasound Room of Functions Branch, Affiliated Hospital of Hebei University, Baoding 071000, China; 9 Department of Thyroid and Breast Surgery, Weihai Municipal Hospital, Cheeloo College of Medicine, Shandong University, Weihai 262400, China; 10 Department of General Surgery, Tianjin Medical University General Hospital, Tianjin 300000, China; 11 Department of Thyroid and Breast Surgery, Tianjin 4th Centre Hospital, Tianjin 300000, China; 12 Department of Thyroid Surgery, Affiliated Hospital of Chengde Medical University, Chengde 067000, China; 13 Department of Pathology, The Second Hospital of Tianjin Medical University, Tianjin 300000, China; 14 Department of Ultrasonography, The Second Hospital of Tianjin Medical University, Tianjin 300000, China; 15 Department of Thyroid, the Second Hospital of Jilin University, Changchun 130041, China; 16 Department of Head and Neck Thyroid Surgery, Cangzhou Hospital of Integrated Traditional Chinese and Western Medicine of Hebei Province, Cangzhou 061000, China; 17 Department of Surgery of Glands, People’s Hospital of Dingzhou, Dingzhou 073000, China; 18 Department of Thyroid and Breast Surgery, Affiliated Hospital of Shaoxing University, Shaoxing 312000, China; 19 Public Laboratory, National Clinical Research Center for Cancer, Key Laboratory of Molecular Cancer Epidemiology of Tianjin, Key Laboratory of Cancer Prevention and Therapy of Tianjin, Tianjin Medical University Cancer Institute and Hospital, Tianjin Medical University, Tianjin 300060, China; 20 Department of Epidemiology and Biostatistics, National Clinical Research Center for Cancer, Key Laboratory of Molecular Cancer Epidemiology of Tianjin, Key Laboratory of Cancer Prevention and Therapy of Tianjin, Tianjin Medical University Cancer Institute and Hospital, Tianjin Medical University, Tianjin 300060, China; 21 Department of Thyroid and Neck Cancer, National Clinical Research Center for Cancer, Key Laboratory of Molecular Cancer Epidemiology of Tianjin, Key Laboratory of Cancer Prevention and Therapy of Tianjin, Tianjin Medical University Cancer Institute and Hospital, Tianjin Medical University, Tianjin 300060, China

**Keywords:** Thyroid cancer, sonographic text report, deep learning, natural language process

## Abstract

**Objective:**

Large volume radiological text data have been accumulated since the incorporation of electronic health record (EHR) systems in clinical practice. We aimed to determine whether deep natural language processing algorithms could aid radiologists in improving thyroid cancer diagnosis.

**Methods:**

Sonographic EHR data were obtained from the EHR database. Pathological reports were used as the gold standard for diagnosing thyroid cancer. We developed thyroid cancer diagnosis based on natural language processing (THCaDxNLP) to interpret unstructured sonographic text reports for thyroid cancer diagnosis. We used the area under the receiver operating characteristic curve (AUROC) as the primary metric to measure the performance of the THCaDxNLP. We compared the performance of thyroid ultrasound radiologists aided with THCaDxNLP *vs*. those without THCaDxNLP using 5 independent test sets.

**Results:**

We obtained a total number of 788,129 sonographic radiological reports. The number of thyroid sonographic data points was 132,277, 18,400 of which were thyroid cancer patients. Among the 5 test sets, the numbers of patients per set were 439, 186, 82, 343, and 171. THCaDxNLP achieved high performance in identifying thyroid cancer patients (the AUROC ranged from 0.857–0.932). Thyroid ultrasound radiologists aided with THCaDxNLP achieved significantly higher performances than those without THCaDxNLP in terms of accuracy (93.8% *vs.* 87.2%; one-sided *t*-test, adjusted *P* = 0.003), precision (92.5% *vs.* 86.0%; *P* = 0.018), and F1 metric (94.2% *vs.* 86.4%; *P* = 0.007).

**Conclusions:**

THCaDxNLP achieved a high AUROC for the identification of thyroid cancer, and improved the accuracy, sensitivity, and precision of thyroid ultrasound radiologists. This warrants further investigation of THCaDxNLP in prospective clinical trials.

## Introduction

Thyroid nodules are prevalently detected in 65% of the general population^1^. The incidence of thyroid cancer has been increasing steadily over the last 2 decades, while its mortality rate has not risen concomitantly^2^. The increase in incidence is likely attributed to over diagnosis conferred by sensitive imaging techniques, especially the increased detection of indolent and well-differentiated forms of thyroid cancer, which eventually leads to unnecessary fine needle aspiration biopsy or thyroidectomy^2^. Thyroid ultrasonographic imaging is a common procedure to screen for thyroid diseases. Interpretation of ultrasonographic results was conducted by radiologists according to the Thyroid Imaging Reporting and Data System (TI-RADS)^3,4^. According to TI-RADS, the clinically relevant ultrasonographic features associated with suspicious malignant phenotypes included solid composition, hypoechogenicity, irregular margins, taller-than-wide shape, and microcalcification. Individuals characterized by these suspicious features should have prompt additional evaluation, whereas those with a cystic or spongiform appearance do not require additional testing^1^.

Deep learning algorithms have been widely used in medical imaging data interpretation, including thyroid cancer diagnosis. The success of deep learning in imaging data understanding has greatly benefitted from the accumulation of large-scale imaging datasets. Recently, we developed a deep learning model on large-scale thyroid ultrasonographic imaging data using pathological reports as the gold standard. In this study, we achieved comparable sensitivity and improved specificity in detecting patients with thyroid cancer, when compared with that from a group of skilled thyroid ultrasonographic radiologists^5^. Beyond the imaging data area, deep learning algorithms have been widely used in natural language processing and modeling. A breakthrough record for language understanding was achieved by a natural language processing algorithm called bidirectional encoder representation from transformers (BERT) on 11 natural language processing tasks^6^. Over the last decade, the accumulation of radiological text data has increased exponentially since the rapid incorporation of electronic health records (EHRs) in routine clinical practice. Improvements in natural language processing algorithms and the accumulation of large-scale data in the EHR system have tremendous potential to transform medical care in diverse scenarios. For example, Liang et al.^7^ developed a deep natural language processing model and demonstrated that it could achieve pediatrician-level accuracy in diagnosing common childhood disease based on massive radiological text reports. Taggart et al.^8^ reported high accuracy of natural language processing algorithms in identifying bleeding events from clinical notes. Kehl et al.^9^ recently demonstrated that deep natural language processing algorithms could predict oncological outcomes from radiological text reports. These studies showed that deep natural language processing algorithms accelerated curation and learning from EHR data.

Chinese word segmentation is a fundamental task in Chinese natural language processing studies. Chinese language sentences are written in a continuous style without an explicit delimiter. The meaning of a single Chinese character is not complete. Thus, it is important to segment Chinese sentences into meaningful words for downstream natural language processing tasks. Ambiguity and unregistered words are 2 major difficulties in Chinese word segmentation. Traditional approaches use character-based sequencing labeling to detect word boundaries within limited and fixed contextual local windows^10,11^. Modern approaches^12–14^ use deep neural networks to model work segmentation tasks by exploring the contextual features.

The purpose of this study was to develop an automated and scalable model that could aid thyroid ultrasonographic radiologists in diagnosing thyroid cancer based on a deep natural language processing algorithm applied to massive radiological text reports. In this study, we first developed a deep natural language processing model called EhrBERT to learn semantic representation within and between sentences of radiological text reports. We next developed thyroid cancer diagnoses based on natural language processing (THCaDxNLP) by fine-tuning the pretrained EhrBERT with thyroid sonographic radiological text reports and corresponding pathological examination results. The performance of THCaDxNLP was comprehensively evaluated using 5 independent test sets. We also compared the performance of radiologists aided with THCaDxNLP *vs.* those without the aid of THCaDxNLP.

## Materials and methods

### Curation of radiological and pathological text report data

We extracted ultrasonographic radiological text reports of adult individuals from the EHR system at Tianjin Medical University Cancer Institute and Hospital (TMUCIH) between January 2012 and December 2017. All radiological text reports of individuals who underwent ultrasonographic examination of the thyroid, neck, and abdomen were included. The pathological examination reports of patients with thyroid disease in the training set were provided by the Pathology Department at TMUCIH. All radiological and pathological text reports were identified before they were transferred to the investigators. This study was approved by the institutional review board of TMUCIH and was conducted in accordance with the Declaration of Helsinki.

### Development and validation of EhrBERT for an understanding of radiological text reports

BERT is an empirically powerful language model that was designed to learn bidirectional representations by jointly conditioning on both left and right sentence contexts^6^. It has achieved state-of-the-art performance on 11 language understanding tasks. The BERT model can address the masked language model (MLM) and next-sentence prediction (NSP) tasks simultaneously. We first tokenized radiological reports by the pretrained lexical analysis model that was specifically developed for the Chinese language^12^. We next built EhrBERT, an EHR language model based on BERT, to jointly learn the semantic representations of radiological text reports. The EhrBERT model was developed for 1 million steps with the Bertadam optimizer, with a batch size of 128, and cosine learning rate decay scheduling with an initial learning rate of 0.0001. We randomly selected 50,000 radiological reports as the validation set. The remaining 1,086,736 records were used to train EhrBERT. The EhrBERT model was trained for 1 million steps. The accuracies of the MLM and NSP of EhrBERT were evaluated on the validation set.

### Development and validation of THCaDxNLP for thyroid cancer diagnosis

We built THCaDxNLP from the pretrained EhrBERT model by adding a classifier at the end of EhrBERT. The weights of the classifier were randomly initialized from a normal distribution with a mean of 0 and a standard deviation of 0.02. The weights of THCaDxNLP were derived from the pre-trained EhrBERT model. We then fine-tuned THCaDxNLP with sonographic radiological reports of thyroid for 8 epochs with the Bertadam optimizer and a cosine learning rate decay scheduling with an initial learning rate of 5e^-5^ and batch size of 16. The total sonographic radiological reports used to develop THCaDxNLP were 172,792, with 142,670 used as the training set and the remaining 30,122 as the validation set. The validation set was used iteratively to test the performance of THCaDxNLP at the end of each training epoch.

### Model ensembling of THCaDxNLP for thyroid cancer (THCA) diagnosis

Model ensembling is a process of combining multiple models and synthesizing the results of each model into a single score to improve the model performance. In this study, we performed model ensembling for the last 5 THCaDxNLP models. We used the area under the receiver operating characteristic curve (AUROC) of these 5 models on the internal validation set as weights to combine the prediction probabilities. Specifically, for a given individual, we denoted the AUROC value of the *i*_th_ model as *w_l_*, and the probability predicted to be THCA by the *i*_th_ model as *P_i_*. The ensemble probabilities from these 5 models were calculated as $P_{\text{ensemble}}=\sum_{i=1}^{5}w_{i}P_{i}$

### Assessment of radiologists aided with THCaDxNLP

Eight thyroid ultrasonographic radiologists were asked to read the radiological text reports of all test sets. We randomly asked 5 thyroid ultrasonographic radiologists (i.e., Q. Zhang, X. Yang, Z. Zheng, Y. Zhao, and L. Liu) to read the radiological text reports of all test sets along with predicted probabilities from THCaDxNLP; they were aware of the AUROC of THCaDxNLP. The remaining 4 radiologists (i.e., J. Zhao, Y. Feng, X. Wang, and J. Li) were asked to read the radiological text reports of all test sets without predicted probabilities from THCaDxNLP. All radiologists read the text reports without any time limit. Each dataset was read by 2 radiologists. The working periods/experience of the radiologists ranged from 6–28 years. The accuracy, sensitivity, specificity, positive predictive rate, negative predictive rate, kappa coefficient, and F1 metric were compared between radiologists with and without THCaDxNLP.

### Statistical analysis

The AUROC was used as the primary metric to measure the performance of the THCaDxNLP. The ROC curve was created by plotting sensitivity and specificity by varying the predicted probability threshold. The F1 metric was calculated as the harmonic average between precision and sensitivity, which was calculated as F1 = 2 × precision × sensitivity/(precision + sensitivity). The 95% confidence intervals for sensitivity, specificity, positive predictive rate, and negative predicted rate were calculated by the Clopper-Pearson method. We plotted ROC and calculated the corresponding AUROC using the R package *pROC,* version 1.10.0 (The R Foundation for Statistical Computing, Vienna, Austria). We calculated the inter-radiologist agreement and Fleiss’ kappa using the R package *irr,* version 0.84. Statistical analysis was conducted using the R software, version 3.4.3. Training and evaluation of EhrBERT and THCaDxNLP were conducted using MXNet, version 1.5.1 and GluonNLP, version 0.8.1 packages. We used precision, recall rate, and F1 score to compare the classification ability of radiologists with and without the aid of THCaDxNLP. A 1-sided *t*-test was used to determine whether radiologists aided with THCaDxNLP achieved higher classification performances than radiologists without THCaDxNLP.

## Results

### A summary of radiological report data

**Figure 1:**
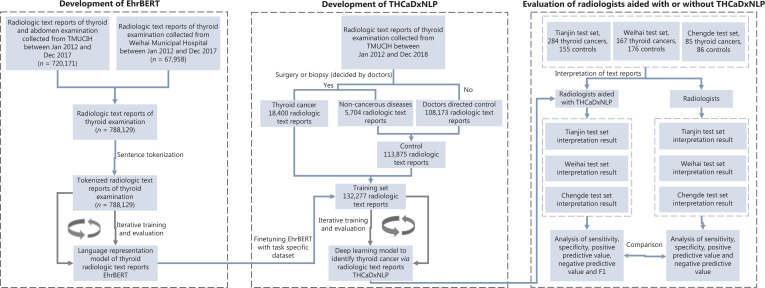
A flowchart depicting the procedures of development and evaluation of EhrBERT for radiological text report modeling and THCaDxNLP for identification of thyroid cancers.

We collected a total of 788,129 ultrasonographic radiological texts from individuals who underwent sonographic examination of the thyroid, breast, and abdomen at TMUCIH and Weihai Municipal Hospital, 132,277 of whom were thyroid sonographic reports, including 18,400 items from THCA patients and 113,875 items from the non-thyroid cancer control group. These text reports were used to develop the EhrBERT model. All THCA patients and 5% (*n* = 5,704) of controls had pathological reports. The remaining 95% of controls (*n* = 108,171) were from outpatient examinations that were deemed benign by sonographic radiologists. A total of 132,277 thyroid sonographic text reports were used as the training set to develop THCaDxNLP. The 5 test sets consisted of 1,221 individuals from TMUCIH (*n* = 439), Tianjin Medical University General Hospital (TGH, *n* = 186), Tianjin Fourth Central Hospital (TFCH, *n* = 82), Weihai Municipal Hospital (Weihai, *n* = 343), and Chengde Hospital (Chengde, *n* = 171). A flowchart depicting this procedure is presented in **Figure 1**. The clinical characteristics of the training set and the 4 test sets are listed in **Supplementary Table S1**.

### The high performance of EhrBERT in sonographic text report modeling

**Figure 2:**
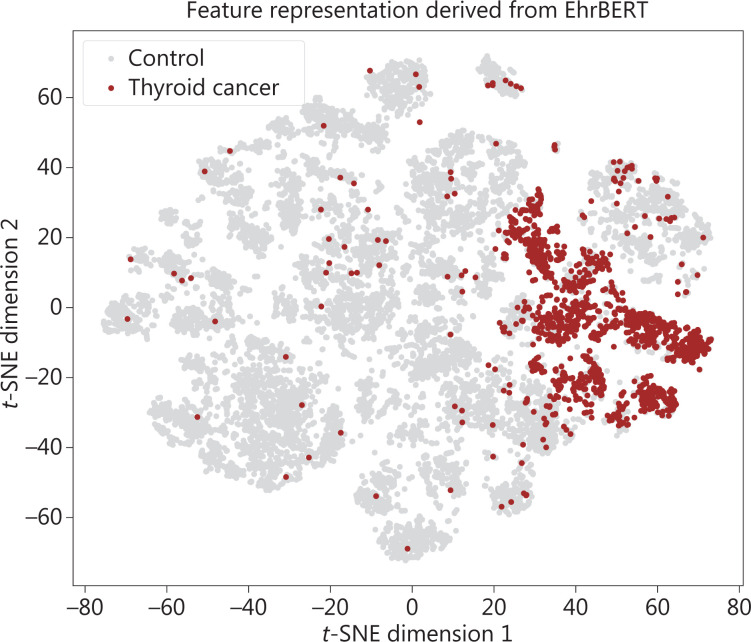
The *t*-SNE visualization of latent features learned by EhrBERT colored by group labels.

We used 14,715 thyroid sonographic text reports to study the latent features learned by EhrBERT. We visualized the latent features learned by EhrBERT using the fast interpolation *t*-SNE projection method^15^. The results showed that the learned representation features of text reports from thyroid cancer patients were distinctive from the control group, although the class label of text reports was not used in the development of EhrBERT (**Figure 2**). The varying loss values of MLM and NSP of EhrBERT are shown in **Supplementary Figure S1**.

### The high performance of THCaDxNLP in identifying THCA

**Figure 3:**
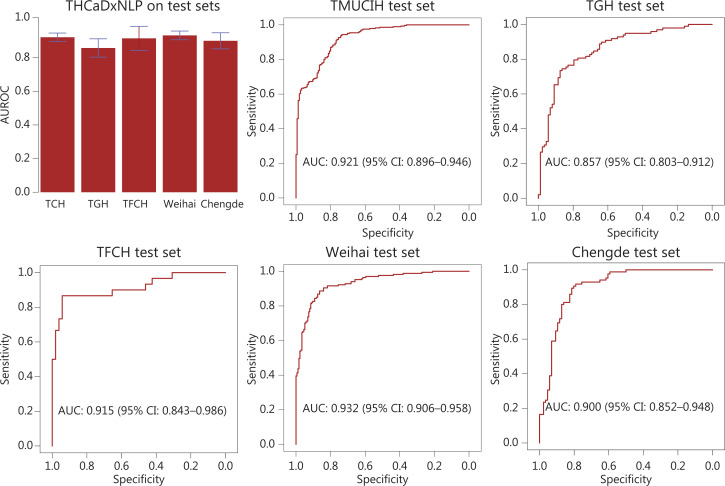
The area under the receiver operating characteristic curve of THCaDxNLP across 5 test sets.

THCaDxNLP achieved an AUROC of 0.921 [95% confidence interval (CI): 0.896–0.946] for the TMUCIH dataset, 0.857 (0.803–0.912) for the TGH dataset, 0.915 (0.843–0.986) for the TFCH dataset, 0.932 (0.906–0.958) for the Weihai dataset, and 0.900 (0.852–0.948) for the Chengde dataset. The ROC curves of THCaDxNLP across the 5 test sets are shown in **Figure 3**. The other classification performance metrics of THCaDxNLP, such as accuracy, sensitivity, positive predictive rate, and F1 score, are provided in **Supplementary Table S2**.

### THCaDxNLP improves the performance of radiologists

**Figure 4:**
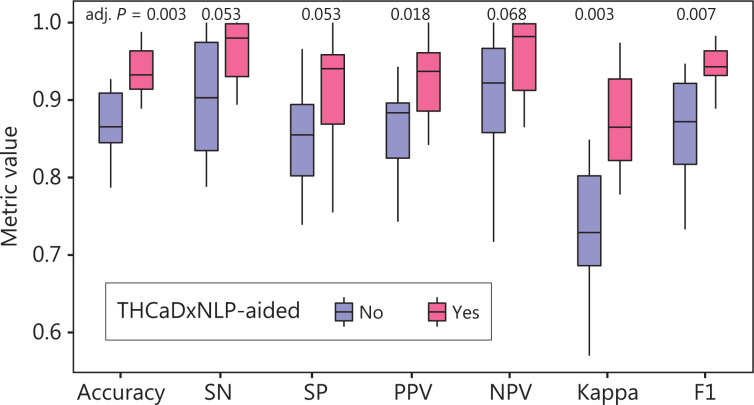
Box plot representation of classification metrics of radiologists aided with THCaDxNLP compared with those without THCaDxNLP. SN is abbreviated for sensitivity, SP for specificity, PPV for positive predictive value, and NPV for negative predictive value.

Thyroid radiologists aided with THCaDxNLP achieved consistent improvements in a variety of classification metrics as compared with those without THCaDxNLP. The detailed performance of radiologists aided with and without THCaDxNLP is shown in **Table 1**. Radiologists aided with THCaDxNLP achieved significantly higher performance on average across these 5 test sets in terms of accuracy (93.8% *vs.* 87.2%; one-sided *t*-test, adjusted *P* = 0.003), positive predictive value (92.5% *vs.* 86.0%; *P* = 0.018), kappa coefficient (87.2% *vs.* 73.6%; *P* = 0.003), and F1 metric (94.2% *vs.* 86.4%; *P* = 0.007). Radiologists aided with THCaDxNLP also showed marginal improvement in sensitivity (96.3% *vs.* 88.2%; *P* = 0.053), specificity (90.7% *vs.* 84.8%; *P* = 0.053), and NPV (95.8% *vs.* 90.5%; *P* = 0.068), although they were not statistically significant (**Figure 4**). In addition, THCaDxNLP improved the radiologists’ inter-rater agreement (0.811 *vs.* 0.776; 1,000 permutations, *P* < 0.001). Across these 5 test sets as a whole, 72 cases were incorrectly interpreted by radiologists without THCaDxNLP, 25 of which were picked by radiologists aided with THCaDxNLP.

**Table 1:** Detailed classification metrics of radiologists with and without THCaDxNLP

Dataset	Radiologist ID	Accuracy (95% CI)	Sensitivity (95% CI)	Specificity (95% CI)	Positive predictive value (95% CI)	Negative predictive value (95% CI)	Kappa^a^	F_1_^b^
**TMUCIH test set (*n* = 439)**	Radiologist 1	0.911 (0.881–0.936)	1.000 (0.990–1.000)	0.748 (0.672–0.815)	0.879 (0.839–0.913)	1.000 (0.975–1.000)	0.794	0.936
	Radiologist 2	0.927 (0.899–0.950)	1.000 (0.990–1.000)	0.794 (0.721–0.854)	0.899 (0.860–0.930)	1.000 (0.976–1.000)	0.833	0.947
	Radiologist 3 aided with THCaDxNLP	0.929 (0.901–0.952)	0.919 (0.881–0.948)	0.948 (0.901–0.977)	0.970 (0.942–0.987)	0.865 (0.804–0.912)	0.849	0.944
	Radiologist 4 aided with THCaDxNLP	0.909 (0.878–0.934)	0.993 (0.975–0.999)	0.755 (0.679–0.820)	0.881 (0.841–0.915)	0.983 (0.941–0.998)	0.789	0.934
**TGH test set (*n* = 186)**	Radiologist 1	0.903 (0.851–0.942)	0.939 (0.871–0.977)	0.864 (0.774–0.928)	0.885 (0.807–0.939)	0.927 (0.848–0.973)	0.805	0.911
	Radiologist 2	0.866 (0.808–0.911)	0.980 (0.928–0.998)	0.739 (0.634–0.827)	0.807 (0.724–0.873)	0.970 (0.896–0.996)	0.727	0.885
	Radiologist 3 aided with THCaDxNLP	0.930 (0.883–0.962)	0.898 (0.820–0.950)	0.966 (0.904–0.993)	0.967 (0.907–0.993)	0.895 (0.815–0.948)	0.86	0.931
	Radiologist 4 aided with THCaDxNLP	0.935 (0.890–0.966)	1.000 (0.970–1.000)	0.864 (0.774–0.928)	0.891 (0.817–0.942)	1.000 (0.961–1.000)	0.87	0.942
**TFCH test set (*n* = 82)**	Radiologist 1	0.841 (0.744–0.913)	0.867 (0.693–0.962)	0.827 (0.697–0.918)	0.743 (0.567–0.875)	0.915 (0.796–0.976)	0.67	0.8
	Radiologist 2	0.854 (0.758–0.922)	0.867 (0.693–0.962)	0.846 (0.719–0.931)	0.765 (0.588–0.893)	0.917 (0.800–0.977)	0.693	0.812
	Radiologist 3 aided with THCaDxNLP	0.976 (0.915–0.997)	1.000 (0.905–1.000)	0.962 (0.868–0.995)	0.938 (0.792–0.992)	1.000 (0.942–1.000)	0.948	0.968
	Radiologist 4 aided with THCaDxNLP	0.988 (0.934–1.000)	0.967 (0.828–0.999)	1.000 (0.944–1.000)	1.000 (0.902–1.000)	0.981 (0.899–1.000)	0.974	0.983
**Weihai test set (*n* = 343)**	Radiologist 1	0.924 (0.891–0.950)	0.958 (0.916–0.983)	0.892 (0.837–0.934)	0.894 (0.839–0.935)	0.957 (0.914–0.983)	0.849	0.925
	Radiologist 2	0.787 (0.740–0.829)	0.599 (0.520–0.674)	0.966 (0.927–0.987)	0.943 (0.881–0.979)	0.717 (0.655–0.774)	0.57	0.733
	Radiologist 3 aided with THCaDxNLP	0.950 (0.922–0.971)	0.964 (0.923–0.987)	0.938 (0.891–0.968)	0.936 (0.888–0.968)	0.965 (0.925–0.987)	0.901	0.95
	Radiologist 4 aided with THCaDxNLP	0.968 (0.943–0.984)	0.994 (0.967–1.000)	0.943 (0.898–0.972)	0.943 (0.898–0.972)	0.994 (0.967–1.000)	0.936	0.968
**Chengde test set (*n* = 171)**	Radiologist 1	0.865 (0.805–0.913)	0.824 (0.726–0.898)	0.907 (0.825–0.959)	0.897 (0.808–0.955)	0.839 (0.748–0.907)	0.731	0.859
	Radiologist 2	0.842 (0.779–0.893)	0.788 (0.686–0.869)	0.895 (0.811–0.951)	0.882 (0.787–0.944)	0.811 (0.717–0.884)	0.684	0.832
	Radiologist 3 aided with THCaDxNLP	0.889 (0.832–0.932)	0.894 (0.808–0.950)	0.884 (0.797–0.943)	0.884 (0.797–0.943)	0.894 (0.808–0.950)	0.778	0.889
	Radiologist 4 aided with THCaDxNLP	0.906 (0.853–0.946)	1.000 (0.965–1.000)	0.814 (0.716–0.890)	0.842 (0.756–0.907)	1.000 (0.958–1.000)	0.813	0.914

^a^Measures the agreement between predicted classification with pathological report. ^b^Harmonic average of the precision and recall rates.

## Discussion

In this study, we developed EhrBERT to obtain semantic representations of radiological text reports and THCaDxNLP to identify thyroid cancer patients by using a deep natural language processing algorithm applied to unstructured EHR data. The performance of THCaDxNLP was evaluated across 5 test sets. A group of 4 thyroid ultrasonographic radiologists aided with THCaDxNLP achieved consistently higher performances than those without THCaDxNLP on a variety of classification metrics. This suggested that THCaDxNLP may be useful as a second reader to help thyroid ultrasonographic radiologists identify THCA patients.

The strength of this study included the development of a text report understanding model with an enormous amount of EHR data, followed by using a fine-tuned, pre-trained model on a task-specific dataset. We developed EhrBERT using 788,129 ultrasonographic text reports. The advantages of pre-training included a good initial point for easier optimization, robustness to overfitting, and more transferrable representations learned from EHR data for downstream tasks^16^. The distinctive *t*-SNE projection of latent feature representation (**Figure 2**) further showed that EhrBERT was able to identify semantic differences between text reports of thyroid cancer patients and controls, even though the classification label of text reports was not included in the development of EhrBERT. This also highlighted the feasibility of fine-tuning EhrBERT for building THCaDxNLP. THCaDxNLP was developed by fine-tuning EhrBERT on thyroid text reports and corresponding labels. This pretraining-then-fine-tuning paradigm in language modeling was demonstrated to be robust to overfitting^16^.

The potential application of THCaDxNLP involves assisting thyroid sonographic radiologists in identifying THCA patients while reducing false positives that would receive unnecessary fine needle aspiration biopsy. This was exemplified by the higher accuracy and precision of thyroid sonographic radiologists aided with or without THCaDxNLP. If integrated into the diagnostic system, THCaDxNLP can instantly provide a second opinion for radiologists. Thyroid sonographic radiologists may find it useful in complex and uncertain cases, which are common in clinical practice. In terms of differential diagnoses, radiologists often make decisions based on their experience, which may bias the cases, based upon those encountered in the past. Because the differential diagnostic ability of THCaDxNLP was learned from a large text report corpus, it would be less biased towards some specific clinical scenarios encountered by an individual radiologist. From this point of view, THCaDxNLP may broaden and augment the differential diagnostic ability of thyroid sonographic radiologists, especially for less experienced radiologists.

A limitation of this study was that the radiological text of each individual in the training and test sets were written by a single radiologist during clinical examination, whose inaccurate interpretation and recording (structured language and unstructured language) could lead to inaccurate interpretation of the second radiologist. In addition, the writing styles of radiological texts varied among radiologists, especially among those from different hospitals. To mitigate the diversity of EHR data, we developed EhrBERT using EHR data from 2 independent medical centers. Nevertheless, the EHR data used to fine-tune EhrBERT to develop THCaDxNLP was only from a single hospital; thus, the diversity of the association between thyroid sonographic text and its corresponding diagnosis could not be fully captured. In the future, we intend to collect more data from more medical centers to further improve the performance of THCaDxNLP. Additionally, most clinical natural language processing tasks, including our study, aimed to link text notes to disease phenotypes but not understand the recognition of relations among entities^17^. Deep learning approaches such as BERT will enable researchers to explore text note understanding. Specifically, the self-attention modules in BERT can provide quantitative relations among different entities. The self-attention module enables the representation of a sentence by attending to a different position in that sentence. The attention score matrix determines the extent of the association among words. Thus, parsing the attention score matrix will contribute to the recognition of entities and clinical conception and may provide a better understanding of clinical natural language processing tasks.

In this study, we developed THCaDxNLP to perform differential diagnoses of THCA based on the clinical expertise of sonographic radiologists, which was complementary to previous studies that analyzed sonographic images to diagnose THCA^5,18^. By analyzing an enormous number of radiological text reports, we could transfer and internalize radiologists’ expertise in deep learning models. This represented a type of transfer of human intelligence to artificial intelligence. In future studies, we plan to integrate THCaDxNLP with the deep learning model learned from thyroid sonographic imaging data. Therefore, human intelligence and machine intelligence will be combined to improve the differential diagnosis of THCA. To what extent THCaDxNLP benefits radiologists remains to be determined, because it depends on the willingness of radiologists to believe and accept artificial intelligence models, which are difficult to quantify. Although EhrBERT and THCaDxNLP were constructed using the Chinese language, the study paradigm in our study could be applied to other languages because the deep learning algorithms used are independent of language. With significant improvements in language translation, it is also feasible to build language-specific EhrBERT and THCaDxNLP models.

## Conclusions

In conclusion, our findings suggested that a deep natural language processing algorithm developed with a large volume of unstructured sonographic EHR data efficiently extracted clinically relevant information associated with THCA from radiological text reports. This technical improvement warrants further investigation of THCaDxNLP in prospective clinical trials to validate its clinical efficacy. If clinically validated, THCaDxNLP can assist sonographic radiologists in the diagnosis of THCA.
