# Supplementary material for: Improved diagnosis of thyroid cancer aided with deep learning applied to sonographic text reports: a retrospective, multi-cohort, diagnostic study
Source: Cancer Biol Med. 2021 Sep 7;19(5):733–41. doi: 10.20892/j.issn.2095-3941.2020.0509 (PMC9196053; doi:10.20892/j.issn.2095-3941.2020.0509)
Supplement: Supplementary file 1 [file cbm-19-733-s001.pdf]

Supplementary materials

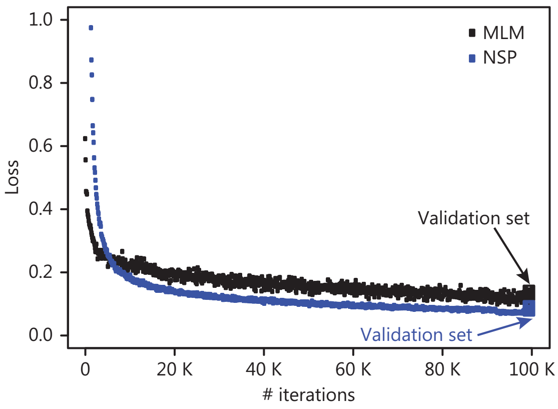

Figure S1 The loss values of the masked language model and next-sentence prediction of EhrBERT.

Table S1 The predicted probability of THCaDxNLP and interpretation of radiologists across 5 test sets

Patient ID	Pathological examination result	Probability predicted to be malignant by THCaDxNLP	Test set	Radiologist1 interpretation without THCaDxNLP	Radiologist2 interpretation without THCaDxNLP	Radiologist3 interpretation with THCaDxNLP	Radiologist4 interpretation with THCaDxNLP
CD1	Malignant	0.9287	Chengde	Benign	Malignant	Malignant	Malignant
CD2	Malignant	0.9902	Chengde	Malignant	Malignant	Malignant	Malignant
CD3	Benign	0.8295	Chengde	Benign	Benign	Malignant	Benign
CD4	Malignant	0.9482	Chengde	Malignant	Malignant	Malignant	Malignant
CD5	Malignant	0.9765	Chengde	Malignant	Malignant	Malignant	Malignant
CD6	Malignant	0.9491	Chengde	Malignant	Malignant	Malignant	Malignant
CD7	Malignant	0.0475	Chengde	Benign	Benign	Malignant	Malignant
CD8	Malignant	0.9666	Chengde	Benign	Benign	Malignant	Malignant
CD9	Malignant	0.9897	Chengde	Benign	Malignant	Malignant	Malignant
CD10	Malignant	0.7730	Chengde	Malignant	Malignant	Benign	Malignant
CD11	Malignant	0.9671	Chengde	Benign	Malignant	Malignant	Malignant
CD12	Malignant	0.9928	Chengde	Malignant	Malignant	Malignant	Malignant
CD13	Malignant	0.9860	Chengde	Malignant	Malignant	Malignant	Malignant
CD14	Malignant	0.9761	Chengde	Malignant	Benign	Malignant	Malignant
CD15	Malignant	0.7920	Chengde	Malignant	Benign	Benign	Malignant
CD16	Malignant	0.9860	Chengde	Malignant	Malignant	Malignant	Malignant
CD17	Malignant	0.8207	Chengde	Malignant	Benign	Benign	Malignant
CD18	Malignant	0.7813	Chengde	Malignant	Benign	Malignant	Malignant
CD19	Malignant	0.1774	Chengde	Malignant	Benign	Malignant	Malignant

Table S1 Continued

Patient ID	Pathological examination result	Probability predicted to be malignant by THCaDxNLP	Test set	Radiologist1 interpretation without THCaDxNLP	Radiologist2 interpretation without THCaDxNLP	Radiologist3 interpretation with THCaDxNLP	Radiologist4 interpretation with THCaDxNLP
CD20	Malignant	0.9856	Chengde	Malignant	Malignant	Malignant	Malignant
CD21	Malignant	0.1936	Chengde	Malignant	Malignant	Malignant	Malignant
CD22	Malignant	0.8163	Chengde	Malignant	Malignant	Malignant	Malignant
CD23	Malignant	0.9822	Chengde	Malignant	Malignant	Malignant	Malignant
CD24	Malignant	0.9812	Chengde	Malignant	Benign	Malignant	Malignant
CD25	Malignant	0.8192	Chengde	Malignant	Malignant	Malignant	Malignant
CD26	Malignant	0.9737	Chengde	Malignant	Malignant	Malignant	Malignant
CD27	Malignant	0.9942	Chengde	Malignant	Malignant	Malignant	Malignant
CD28	Malignant	0.9464	Chengde	Malignant	Malignant	Malignant	Malignant
CD29	Benign	0.9901	Chengde	Benign	Benign	Malignant	Malignant
CD30	Malignant	0.9818	Chengde	Malignant	Benign	Malignant	Malignant
CD31	Malignant	0.9923	Chengde	Malignant	Benign	Malignant	Malignant
CD32	Malignant	0.8989	Chengde	Malignant	Malignant	Malignant	Malignant
CD33	Malignant	0.9054	Chengde	Malignant	Malignant	Malignant	Malignant
CD34	Malignant	0.9651	Chengde	Malignant	Malignant	Malignant	Malignant
CD35	Malignant	0.8142	Chengde	Malignant	Malignant	Benign	Malignant
CD36	Malignant	0.9917	Chengde	Malignant	Malignant	Malignant	Malignant
CD37	Malignant	0.9586	Chengde	Malignant	Malignant	Malignant	Malignant
CD38	Malignant	0.9915	Chengde	Malignant	Malignant	Malignant	Malignant
CD39	Malignant	0.9895	Chengde	Malignant	Malignant	Malignant	Malignant
CD40	Malignant	0.3921	Chengde	Benign	Benign	Benign	Malignant
CD41	Malignant	0.9341	Chengde	Malignant	Malignant	Malignant	Malignant
CD42	Malignant	0.5784	Chengde	Benign	Benign	Malignant	Malignant
CD43	Malignant	0.9787	Chengde	Malignant	Malignant	Malignant	Malignant
CD44	Malignant	0.9869	Chengde	Malignant	Malignant	Malignant	Malignant
CD45	Malignant	0.9894	Chengde	Malignant	Malignant	Malignant	Malignant
CD46	Malignant	0.8936	Chengde	Malignant	Malignant	Malignant	Malignant
CD47	Malignant	0.9638	Chengde	Malignant	Malignant	Malignant	Malignant
CD48	Malignant	0.8891	Chengde	Benign	Benign	Malignant	Malignant
CD49	Malignant	0.8961	Chengde	Benign	Benign	Benign	Malignant
CD50	Malignant	0.9876	Chengde	Malignant	Malignant	Malignant	Malignant
CD51	Malignant	0.8564	Chengde	Malignant	Malignant	Malignant	Malignant
CD52	Benign	0.0061	Chengde	Benign	Malignant	Malignant	Malignant

Table S1 Continued

Patient ID	Pathological examination result	Probability predicted to be malignant by THCaDxNLP	Test set	Radiologist1 interpretation without THCaDxNLP	Radiologist2 interpretation without THCaDxNLP	Radiologist3 interpretation with THCaDxNLP	Radiologist4 interpretation with THCaDxNLP
CD53	Malignant	0.9853	Chengde	Malignant	Malignant	Malignant	Malignant
CD54	Malignant	0.9932	Chengde	Malignant	Malignant	Malignant	Malignant
CD55	Malignant	0.9208	Chengde	Malignant	Malignant	Malignant	Malignant
CD56	Malignant	0.9295	Chengde	Malignant	Malignant	Malignant	Malignant
CD57	Malignant	0.9810	Chengde	Malignant	Malignant	Malignant	Malignant
CD58	Malignant	0.9823	Chengde	Malignant	Malignant	Malignant	Malignant
CD59	Malignant	0.7965	Chengde	Malignant	Malignant	Malignant	Malignant
CD60	Malignant	0.9872	Chengde	Malignant	Malignant	Malignant	Malignant
CD61	Malignant	0.9811	Chengde	Malignant	Malignant	Malignant	Malignant
CD62	Malignant	0.9922	Chengde	Malignant	Malignant	Malignant	Malignant
CD63	Malignant	0.9589	Chengde	Malignant	Benign	Malignant	Malignant
CD64	Malignant	0.9850	Chengde	Malignant	Malignant	Malignant	Malignant
CD65	Malignant	0.9944	Chengde	Malignant	Malignant	Malignant	Malignant
CD66	Malignant	0.9392	Chengde	Malignant	Malignant	Benign	Malignant
CD67	Malignant	0.9484	Chengde	Benign	Malignant	Malignant	Malignant
CD68	Malignant	0.9858	Chengde	Malignant	Malignant	Malignant	Malignant
CD69	Malignant	0.9666	Chengde	Malignant	Malignant	Malignant	Malignant
CD70	Malignant	0.9583	Chengde	Benign	Malignant	Malignant	Malignant
CD71	Malignant	0.9711	Chengde	Malignant	Benign	Malignant	Malignant
CD72	Malignant	0.0523	Chengde	Benign	Malignant	Malignant	Malignant
CD73	Malignant	0.9930	Chengde	Malignant	Malignant	Malignant	Malignant
CD74	Malignant	0.9949	Chengde	Malignant	Malignant	Malignant	Malignant
CD75	Malignant	0.0179	Chengde	Benign	Benign	Benign	Malignant
CD76	Malignant	0.9890	Chengde	Malignant	Malignant	Malignant	Malignant
CD77	Malignant	0.8918	Chengde	Malignant	Malignant	Malignant	Malignant
CD78	Malignant	0.9323	Chengde	Benign	Benign	Benign	Malignant
CD79	Malignant	0.9716	Chengde	Malignant	Malignant	Malignant	Malignant
CD80	Malignant	0.9751	Chengde	Malignant	Malignant	Malignant	Malignant
CD81	Malignant	0.9760	Chengde	Malignant	Malignant	Malignant	Malignant
CD82	Malignant	0.9933	Chengde	Malignant	Malignant	Malignant	Malignant
CD83	Malignant	0.9912	Chengde	Benign	Malignant	Malignant	Malignant
CD84	Malignant	0.0432	Chengde	Malignant	Malignant	Malignant	Malignant
CD85	Malignant	0.9274	Chengde	Malignant	Malignant	Malignant	Malignant

Table S1 Continued

Patient ID	Pathological examination result	Probability predicted to be malignant by THCaDxNLP	Test set	Radiologist1 interpretation without THCaDxNLP	Radiologist2 interpretation without THCaDxNLP	Radiologist3 interpretation with THCaDxNLP	Radiologist4 interpretation with THCaDxNLP
CD86	Malignant	0.9629	Chengde	Malignant	Malignant	Malignant	Malignant
CD87	Malignant	0.8871	Chengde	Malignant	Malignant	Malignant	Malignant
CD88	Benign	0.0071	Chengde	Benign	Benign	Benign	Malignant
CD89	Malignant	0.9908	Chengde	Malignant	Malignant	Malignant	Malignant
CD90	Benign	0.1873	Chengde	Benign	Benign	Benign	Benign
CD91	Benign	0.8125	Chengde	Benign	Malignant	Malignant	Malignant
CD92	Benign	0.0058	Chengde	Benign	Benign	Benign	Benign
CD93	Benign	0.0013	Chengde	Benign	Benign	Benign	Benign
CD94	Benign	0.0024	Chengde	Benign	Benign	Benign	Benign
CD95	Benign	0.0015	Chengde	Benign	Benign	Benign	Benign
CD96	Benign	0.2164	Chengde	Benign	Benign	Malignant	Benign
CD97	Benign	0.0051	Chengde	Benign	Benign	Benign	Benign
CD98	Benign	0.6505	Chengde	Benign	Benign	Benign	Benign
CD99	Benign	0.0019	Chengde	Benign	Benign	Benign	Benign
CD100	Benign	0.0023	Chengde	Benign	Benign	Benign	Benign
CD101	Benign	0.0206	Chengde	Benign	Benign	Benign	Benign
CD102	Benign	0.9865	Chengde	Benign	Benign	Benign	Malignant
CD103	Benign	0.0039	Chengde	Benign	Benign	Benign	Benign
CD104	Benign	0.0044	Chengde	Benign	Benign	Benign	Benign
CD105	Benign	0.0468	Chengde	Benign	Benign	Malignant	Benign
CD106	Benign	0.8237	Chengde	Benign	Benign	Benign	Benign
CD107	Benign	0.9551	Chengde	Malignant	Benign	Malignant	Malignant
CD108	Benign	0.0015	Chengde	Benign	Benign	Benign	Benign
CD109	Benign	0.0025	Chengde	Benign	Benign	Benign	Benign
CD110	Benign	0.0035	Chengde	Benign	Benign	Benign	Benign
CD111	Benign	0.0028	Chengde	Benign	Benign	Benign	Benign
CD112	Benign	0.0051	Chengde	Benign	Benign	Benign	Benign
CD113	Benign	0.3920	Chengde	Benign	Benign	Benign	Benign
CD114	Benign	0.0114	Chengde	Benign	Benign	Benign	Benign
CD115	Benign	0.0013	Chengde	Benign	Benign	Benign	Benign
CD116	Benign	0.2145	Chengde	Benign	Benign	Benign	Benign
CD117	Benign	0.0073	Chengde	Benign	Benign	Benign	Benign
CD118	Benign	0.0066	Chengde	Benign	Benign	Benign	Benign

Table S1 Continued

Patient ID	Pathological examination result	Probability predicted to be malignant by THCaDxNLP	Test set	Radiologist1 interpretation without THCaDxNLP	Radiologist2 interpretation without THCaDxNLP	Radiologist3 interpretation with THCaDxNLP	Radiologist4 interpretation with THCaDxNLP
CD119	Benign	0.0024	Chengde	Benign	Benign	Benign	Benign
CD120	Benign	0.0370	Chengde	Benign	Benign	Benign	Benign
CD121	Benign	0.9851	Chengde	Malignant	Malignant	Benign	Malignant
CD122	Benign	0.0012	Chengde	Benign	Benign	Benign	Benign
CD123	Benign	0.0184	Chengde	Benign	Benign	Benign	Benign
CD124	Benign	0.9806	Chengde	Malignant	Malignant	Benign	Malignant
CD125	Benign	0.0156	Chengde	Benign	Benign	Benign	Benign
CD126	Benign	0.0074	Chengde	Benign	Benign	Benign	Benign
CD127	Benign	0.0071	Chengde	Benign	Benign	Benign	Benign
CD128	Benign	0.0020	Chengde	Benign	Benign	Benign	Benign
CD129	Benign	0.0060	Chengde	Benign	Benign	Benign	Benign
CD130	Benign	0.9280	Chengde	Malignant	Malignant	Benign	Malignant
CD131	Benign	0.0241	Chengde	Benign	Benign	Benign	Benign
CD132	Benign	0.0052	Chengde	Benign	Benign	Benign	Benign
CD133	Benign	0.0117	Chengde	Benign	Benign	Benign	Benign
CD134	Benign	0.0183	Chengde	Benign	Benign	Benign	Benign
CD135	Benign	0.2026	Chengde	Benign	Benign	Benign	Benign
CD136	Benign	0.0014	Chengde	Benign	Benign	Benign	Benign
CD137	Benign	0.1947	Chengde	Benign	Benign	Benign	Malignant
CD138	Benign	0.0082	Chengde	Benign	Benign	Benign	Benign
CD139	Benign	0.0182	Chengde	Benign	Benign	Benign	Benign
CD140	Benign	0.0093	Chengde	Benign	Benign	Benign	Benign
CD141	Benign	0.9869	Chengde	Malignant	Benign	Malignant	Malignant
CD142	Benign	0.0050	Chengde	Benign	Benign	Benign	Benign
CD143	Benign	0.2265	Chengde	Benign	Benign	Benign	Malignant
CD144	Benign	0.0059	Chengde	Benign	Benign	Benign	Benign
CD145	Benign	0.4219	Chengde	Benign	Benign	Benign	Benign
CD146	Benign	0.0094	Chengde	Benign	Benign	Benign	Benign
CD147	Benign	0.9901	Chengde	Malignant	Malignant	Benign	Malignant
CD148	Benign	0.9536	Chengde	Benign	Benign	Benign	Malignant
CD149	Benign	0.0430	Chengde	Benign	Malignant	Malignant	Benign
CD150	Benign	0.9176	Chengde	Benign	Benign	Benign	Benign
CD151	Benign	0.0040	Chengde	Benign	Benign	Benign	Benign

Table S1 Continued

Patient ID	Pathological examination result	Probability predicted to be malignant by THCaDxNLP	Test set	Radiologist1 interpretation without THCaDxNLP	Radiologist2 interpretation without THCaDxNLP	Radiologist3 interpretation with THCaDxNLP	Radiologist4 interpretation with THCaDxNLP
CD152	Benign	0.1794	Chengde	Benign	Benign	Benign	Benign
CD153	Benign	0.0024	Chengde	Benign	Benign	Benign	Benign
CD154	Benign	0.0052	Chengde	Benign	Benign	Benign	Benign
CD155	Benign	0.5577	Chengde	Malignant	Malignant	Benign	Malignant
CD156	Benign	0.7776	Chengde	Benign	Benign	Benign	Benign
CD157	Benign	0.1292	Chengde	Benign	Benign	Benign	Benign
CD158	Benign	0.0054	Chengde	Benign	Benign	Benign	Benign
CD159	Benign	0.3619	Chengde	Benign	Benign	Benign	Benign
CD160	Benign	0.2154	Chengde	Benign	Benign	Benign	Benign
CD161	Benign	0.0061	Chengde	Benign	Benign	Benign	Benign
CD162	Benign	0.9351	Chengde	Malignant	Malignant	Malignant	Benign
CD163	Benign	0.8789	Chengde	Benign	Benign	Benign	Malignant
CD164	Benign	0.0036	Chengde	Benign	Benign	Benign	Benign
CD165	Benign	0.5278	Chengde	Benign	Benign	Benign	Benign
CD166	Benign	0.0021	Chengde	Benign	Benign	Benign	Benign
CD167	Benign	0.1825	Chengde	Benign	Benign	Benign	Benign
CD168	Benign	0.0231	Chengde	Benign	Benign	Benign	Benign
CD169	Benign	0.0032	Chengde	Benign	Benign	Benign	Benign
CD170	Benign	0.8296	Chengde	Benign	Benign	Benign	Benign
CD171	Benign	0.3878	Chengde	Benign	Benign	Benign	Benign
TJ1	Benign	0.9754	Tianjin	Benign	Benign	Benign	Benign
TJ2	Benign	0.0294	Tianjin	Benign	Benign	Benign	Benign
TJ3	Benign	0.9284	Tianjin	Benign	Benign	Benign	Benign
TJ4	Benign	0.0010	Tianjin	Benign	Benign	Benign	Benign
TJ5	Benign	0.0240	Tianjin	Benign	Benign	Benign	Benign
TJ6	Benign	0.9422	Tianjin	Malignant	Malignant	Benign	Malignant
TJ7	Benign	0.0034	Tianjin	Benign	Benign	Benign	Benign
TJ8	Benign	0.0021	Tianjin	Benign	Benign	Benign	Benign
TJ9	Benign	0.9433	Tianjin	Malignant	Benign	Benign	Malignant
TJ10	Benign	0.0031	Tianjin	Benign	Benign	Benign	Benign
TJ11	Benign	0.0047	Tianjin	Benign	Benign	Benign	Benign
TJ12	Benign	0.3746	Tianjin	Benign	Benign	Benign	Benign
TJ13	Benign	0.0049	Tianjin	Benign	Benign	Benign	Benign

Table S1 Continued

Patient ID	Pathological examination result	Probability predicted to be malignant by THCaDxNLP	Test set	Radiologist1 interpretation without THCaDxNLP	Radiologist2 interpretation without THCaDxNLP	Radiologist3 interpretation with THCaDxNLP	Radiologist4 interpretation with THCaDxNLP
TJ14	Benign	0.9315	Tianjin	Malignant	Malignant	Benign	Malignant
TJ15	Benign	0.0040	Tianjin	Benign	Benign	Benign	Benign
TJ16	Benign	0.2725	Tianjin	Benign	Benign	Malignant	Malignant
TJ17	Benign	0.9316	Tianjin	Malignant	Benign	Benign	Malignant
TJ18	Benign	0.8465	Tianjin	Benign	Benign	Benign	Malignant
TJ19	Benign	0.0040	Tianjin	Benign	Benign	Benign	Benign
TJ20	Benign	0.0006	Tianjin	Benign	Benign	Benign	Benign
TJ21	Benign	0.9450	Tianjin	Benign	Benign	Malignant	Malignant
TJ22	Benign	0.0041	Tianjin	Benign	Benign	Benign	Benign
TJ23	Benign	0.0005	Tianjin	Benign	Benign	Benign	Benign
TJ24	Benign	0.0006	Tianjin	Benign	Benign	Benign	Benign
TJ25	Benign	0.0029	Tianjin	Benign	Benign	Benign	Benign
TJ26	Benign	0.0007	Tianjin	Benign	Benign	Benign	Benign
TJ27	Benign	0.0070	Tianjin	Malignant	Malignant	Benign	Malignant
TJ28	Benign	0.0084	Tianjin	Benign	Benign	Benign	Benign
TJ29	Benign	0.9197	Tianjin	Malignant	Benign	Benign	Benign
TJ30	Benign	0.0008	Tianjin	Benign	Benign	Benign	Benign
TJ31	Benign	0.9602	Tianjin	Benign	Benign	Malignant	Malignant
TJ32	Benign	0.0059	Tianjin	Benign	Benign	Benign	Benign
TJ33	Benign	0.0111	Tianjin	Benign	Benign	Benign	Benign
TJ34	Benign	0.0005	Tianjin	Benign	Benign	Benign	Benign
TJ35	Benign	0.0014	Tianjin	Benign	Benign	Benign	Benign
TJ36	Benign	0.0096	Tianjin	Benign	Benign	Benign	Benign
TJ37	Benign	0.8825	Tianjin	Malignant	Malignant	Benign	Malignant
TJ38	Benign	0.0013	Tianjin	Benign	Benign	Benign	Benign
TJ39	Benign	0.0120	Tianjin	Benign	Benign	Benign	Benign
TJ40	Benign	0.0046	Tianjin	Benign	Benign	Benign	Benign
TJ41	Benign	0.9371	Tianjin	Malignant	Malignant	Benign	Malignant
TJ42	Benign	0.0009	Tianjin	Benign	Benign	Benign	Benign
TJ43	Benign	0.9018	Tianjin	Malignant	Malignant	Benign	Malignant
TJ44	Benign	0.9083	Tianjin	Benign	Benign	Malignant	Benign
TJ45	Benign	0.0505	Tianjin	Benign	Benign	Benign	Benign
TJ46	Benign	0.0043	Tianjin	Benign	Benign	Benign	Benign

Table S1 Continued

Patient ID	Pathological examination result	Probability predicted to be malignant by THCaDxNLP	Test set	Radiologist1 interpretation without THCaDxNLP	Radiologist2 interpretation without THCaDxNLP	Radiologist3 interpretation with THCaDxNLP	Radiologist4 interpretation with THCaDxNLP
TJ47	Benign	0.0058	Tianjin	Benign	Benign	Benign	Benign
TJ48	Benign	0.0105	Tianjin	Benign	Benign	Benign	Benign
TJ49	Benign	0.8711	Tianjin	Benign	Benign	Benign	Malignant
TJ50	Benign	0.0585	Tianjin	Benign	Benign	Benign	Benign
TJ51	Benign	0.0039	Tianjin	Benign	Benign	Benign	Benign
TJ52	Benign	0.9473	Tianjin	Benign	Benign	Malignant	Malignant
TJ53	Benign	0.0005	Tianjin	Benign	Benign	Benign	Benign
TJ54	Benign	0.0206	Tianjin	Benign	Benign	Benign	Benign
TJ55	Benign	0.3360	Tianjin	Malignant	Malignant	Benign	Benign
TJ56	Benign	0.0166	Tianjin	Benign	Benign	Benign	Benign
TJ57	Benign	0.0071	Tianjin	Benign	Benign	Benign	Benign
TJ58	Benign	0.0097	Tianjin	Benign	Benign	Benign	Benign
TJ59	Benign	0.7726	Tianjin	Malignant	Malignant	Benign	Malignant
TJ60	Benign	0.0092	Tianjin	Benign	Benign	Benign	Benign
TJ61	Benign	0.0062	Tianjin	Benign	Benign	Benign	Benign
TJ62	Benign	0.0060	Tianjin	Malignant	Benign	Benign	Benign
TJ63	Benign	0.8842	Tianjin	Malignant	Malignant	Malignant	Benign
TJ64	Benign	0.0660	Tianjin	Benign	Benign	Benign	Benign
TJ65	Benign	0.0082	Tianjin	Benign	Benign	Benign	Benign
TJ66	Benign	0.0053	Tianjin	Benign	Benign	Benign	Benign
TJ67	Benign	0.0140	Tianjin	Benign	Benign	Benign	Benign
TJ68	Benign	0.0018	Tianjin	Benign	Benign	Benign	Benign
TJ69	Benign	0.0011	Tianjin	Benign	Benign	Benign	Benign
TJ70	Benign	0.2976	Tianjin	Benign	Benign	Benign	Benign
TJ71	Benign	0.9023	Tianjin	Malignant	Malignant	Benign	Malignant
TJ72	Benign	0.0099	Tianjin	Benign	Benign	Benign	Benign
TJ73	Benign	0.0316	Tianjin	Benign	Benign	Benign	Benign
TJ74	Benign	0.9338	Tianjin	Malignant	Malignant	Benign	Malignant
TJ75	Benign	0.0133	Tianjin	Benign	Benign	Benign	Benign
TJ76	Benign	0.0049	Tianjin	Benign	Benign	Benign	Benign
TJ77	Benign	0.0005	Tianjin	Benign	Benign	Benign	Benign
TJ78	Benign	0.0026	Tianjin	Benign	Benign	Benign	Benign
TJ79	Benign	0.0312	Tianjin	Benign	Benign	Benign	Benign

Table S1 Continued

Patient ID	Pathological examination result	Probability predicted to be malignant by THCaDxNLP	Test set	Radiologist1 interpretation without THCaDxNLP	Radiologist2 interpretation without THCaDxNLP	Radiologist3 interpretation with THCaDxNLP	Radiologist4 interpretation with THCaDxNLP
TJ80	Benign	0.0125	Tianjin	Benign	Benign	Benign	Benign
TJ81	Benign	0.0619	Tianjin	Benign	Benign	Benign	Benign
TJ82	Benign	0.0460	Tianjin	Benign	Benign	Benign	Malignant
TJ83	Benign	0.0087	Tianjin	Benign	Benign	Benign	Benign
TJ84	Benign	0.0527	Tianjin	Benign	Benign	Benign	Benign
TJ85	Benign	0.0065	Tianjin	Benign	Benign	Benign	Benign
TJ86	Benign	0.0089	Tianjin	Benign	Benign	Benign	Benign
TJ87	Benign	0.9275	Tianjin	Malignant	Malignant	Benign	Malignant
TJ88	Benign	0.9426	Tianjin	Malignant	Malignant	Benign	Malignant
TJ89	Benign	0.0260	Tianjin	Benign	Benign	Benign	Benign
TJ90	Benign	0.0558	Tianjin	Benign	Benign	Benign	Benign
TJ91	Benign	0.0081	Tianjin	Benign	Benign	Benign	Benign
TJ92	Benign	0.0005	Tianjin	Benign	Benign	Benign	Benign
TJ93	Benign	0.0032	Tianjin	Benign	Benign	Benign	Benign
TJ94	Benign	0.5852	Tianjin	Malignant	Malignant	Benign	Malignant
TJ95	Benign	0.0006	Tianjin	Benign	Benign	Benign	Benign
TJ96	Benign	0.0048	Tianjin	Benign	Benign	Benign	Benign
TJ97	Benign	0.0026	Tianjin	Benign	Benign	Benign	Benign
TJ98	Benign	0.0034	Tianjin	Benign	Benign	Benign	Benign
TJ99	Benign	0.4295	Tianjin	Malignant	Malignant	Benign	Malignant
TJ100	Benign	0.9520	Tianjin	Benign	Benign	Malignant	Malignant
TJ101	Benign	0.0025	Tianjin	Benign	Benign	Benign	Benign
TJ102	Benign	0.0558	Tianjin	Benign	Benign	Benign	Benign
TJ103	Benign	0.9327	Tianjin	Malignant	Benign	Malignant	Malignant
TJ104	Benign	0.7927	Tianjin	Benign	Benign	Benign	Malignant
TJ105	Benign	0.9049	Tianjin	Malignant	Malignant	Benign	Malignant
TJ106	Benign	0.0108	Tianjin	Benign	Benign	Benign	Benign
TJ107	Benign	0.0381	Tianjin	Malignant	Benign	Benign	Malignant
TJ108	Benign	0.0755	Tianjin	Benign	Benign	Benign	Benign
TJ109	Benign	0.0291	Tianjin	Malignant	Malignant	Benign	Benign
TJ110	Benign	0.0159	Tianjin	Benign	Benign	Benign	Benign
TJ111	Benign	0.9301	Tianjin	Malignant	Malignant	Benign	Malignant
TJ112	Benign	0.0061	Tianjin	Benign	Benign	Benign	Benign

Table S1 Continued

Patient ID	Pathological examination result	Probability predicted to be malignant by THCaDxNLP	Test set	Radiologist1 interpretation without THCaDxNLP	Radiologist2 interpretation without THCaDxNLP	Radiologist3 interpretation with THCaDxNLP	Radiologist4 interpretation with THCaDxNLP
TJ113	Benign	0.0057	Tianjin	Benign	Benign	Benign	Benign
TJ114	Benign	0.0014	Tianjin	Benign	Benign	Benign	Benign
TJ115	Benign	0.7603	Tianjin	Malignant	Malignant	Benign	Benign
TJ116	Benign	0.0098	Tianjin	Benign	Benign	Benign	Benign
TJ117	Benign	0.0005	Tianjin	Benign	Benign	Benign	Benign
TJ118	Benign	0.3371	Tianjin	Malignant	Malignant	Benign	Benign
TJ119	Benign	0.0008	Tianjin	Benign	Benign	Benign	Benign
TJ120	Benign	0.0075	Tianjin	Benign	Benign	Benign	Benign
TJ121	Benign	0.0081	Tianjin	Benign	Benign	Benign	Benign
TJ122	Benign	0.0075	Tianjin	Benign	Benign	Benign	Benign
TJ123	Benign	0.0075	Tianjin	Benign	Benign	Benign	Benign
TJ124	Benign	0.1296	Tianjin	Benign	Benign	Benign	Benign
TJ125	Benign	0.0068	Tianjin	Malignant	Benign	Benign	Malignant
TJ126	Benign	0.0006	Tianjin	Benign	Benign	Benign	Benign
TJ127	Benign	0.4147	Tianjin	Malignant	Malignant	Benign	Malignant
TJ128	Benign	0.0415	Tianjin	Benign	Benign	Benign	Benign
TJ129	Benign	0.9213	Tianjin	Malignant	Malignant	Benign	Malignant
TJ130	Benign	0.0064	Tianjin	Benign	Benign	Benign	Benign
TJ131	Benign	0.0931	Tianjin	Malignant	Malignant	Benign	Malignant
TJ132	Benign	0.8838	Tianjin	Malignant	Malignant	Benign	Malignant
TJ133	Benign	0.1249	Tianjin	Malignant	Malignant	Benign	Malignant
TJ134	Benign	0.0292	Tianjin	Benign	Benign	Benign	Benign
TJ135	Benign	0.0022	Tianjin	Benign	Benign	Benign	Benign
TJ136	Benign	0.0040	Tianjin	Malignant	Malignant	Benign	Benign
TJ137	Benign	0.0061	Tianjin	Benign	Benign	Benign	Benign
TJ138	Benign	0.8029	Tianjin	Benign	Benign	Benign	Benign
TJ139	Benign	0.0206	Tianjin	Benign	Benign	Benign	Benign
TJ140	Benign	0.0194	Tianjin	Benign	Benign	Benign	Benign
TJ141	Benign	0.5773	Tianjin	Malignant	Malignant	Benign	Benign
TJ142	Benign	0.0163	Tianjin	Benign	Benign	Benign	Benign
TJ143	Benign	0.9417	Tianjin	Malignant	Malignant	Benign	Malignant
TJ144	Benign	0.0012	Tianjin	Benign	Benign	Benign	Benign
TJ145	Benign	0.9414	Tianjin	Malignant	Malignant	Benign	Malignant

Table S1 Continued

Patient ID	Pathological examination result	Probability predicted to be malignant by THCaDxNLP	Test set	Radiologist1 interpretation without THCaDxNLP	Radiologist2 interpretation without THCaDxNLP	Radiologist3 interpretation with THCaDxNLP	Radiologist4 interpretation with THCaDxNLP
TJ146	Benign	0.6999	Tianjin	Benign	Benign	Benign	Benign
TJ147	Benign	0.0202	Tianjin	Benign	Benign	Benign	Benign
TJ148	Benign	0.0009	Tianjin	Benign	Benign	Benign	Benign
TJ149	Benign	0.3555	Tianjin	Malignant	Malignant	Benign	Benign
TJ150	Benign	0.0093	Tianjin	Benign	Benign	Benign	Benign
TJ151	Benign	0.9284	Tianjin	Malignant	Malignant	Benign	Malignant
TJ152	Benign	0.0060	Tianjin	Benign	Benign	Benign	Benign
TJ153	Benign	0.0175	Tianjin	Benign	Benign	Benign	Benign
TJ154	Benign	0.0024	Tianjin	Benign	Benign	Benign	Benign
TJ155	Benign	0.8937	Tianjin	Malignant	Malignant	Benign	Malignant
TJ156	Malignant	0.8998	Tianjin	Malignant	Malignant	Malignant	Malignant
TJ157	Malignant	0.9329	Tianjin	Malignant	Malignant	Malignant	Malignant
TJ158	Malignant	0.0417	Tianjin	Malignant	Malignant	Malignant	Malignant
TJ159	Malignant	0.9022	Tianjin	Malignant	Malignant	Malignant	Malignant
TJ160	Malignant	0.9771	Tianjin	Malignant	Malignant	Malignant	Malignant
TJ161	Malignant	0.9694	Tianjin	Malignant	Malignant	Malignant	Malignant
TJ162	Malignant	0.9871	Tianjin	Malignant	Malignant	Malignant	Malignant
TJ163	Malignant	0.9647	Tianjin	Malignant	Malignant	Benign	Malignant
TJ164	Malignant	0.9784	Tianjin	Malignant	Malignant	Malignant	Malignant
TJ165	Malignant	0.7810	Tianjin	Malignant	Malignant	Malignant	Malignant
TJ166	Malignant	0.9682	Tianjin	Malignant	Malignant	Malignant	Malignant
TJ167	Malignant	0.9859	Tianjin	Malignant	Malignant	Malignant	Malignant
TJ168	Malignant	0.9470	Tianjin	Malignant	Malignant	Malignant	Malignant
TJ169	Malignant	0.9817	Tianjin	Malignant	Malignant	Malignant	Malignant
TJ170	Malignant	0.9315	Tianjin	Malignant	Malignant	Malignant	Malignant
TJ171	Malignant	0.9592	Tianjin	Malignant	Malignant	Malignant	Malignant
TJ172	Malignant	0.9753	Tianjin	Malignant	Malignant	Malignant	Malignant
TJ173	Malignant	0.9367	Tianjin	Malignant	Malignant	Malignant	Malignant
TJ174	Malignant	0.0156	Tianjin	Malignant	Malignant	Malignant	Malignant
TJ175	Malignant	0.9754	Tianjin	Malignant	Malignant	Malignant	Malignant
TJ176	Malignant	0.9612	Tianjin	Malignant	Malignant	Malignant	Malignant
TJ177	Malignant	0.9758	Tianjin	Malignant	Malignant	Malignant	Malignant
TJ178	Malignant	0.9506	Tianjin	Malignant	Malignant	Malignant	Malignant

Table S1 Continued

Patient ID	Pathological examination result	Probability predicted to be malignant by THCaDxNLP	Test set	Radiologist1 interpretation without THCaDxNLP	Radiologist2 interpretation without THCaDxNLP	Radiologist3 interpretation with THCaDxNLP	Radiologist4 interpretation with THCaDxNLP
TJ179	Malignant	0.9637	Tianjin	Malignant	Malignant	Malignant	Malignant
TJ180	Malignant	0.8244	Tianjin	Malignant	Malignant	Malignant	Malignant
TJ181	Malignant	0.9883	Tianjin	Malignant	Malignant	Malignant	Malignant
TJ182	Malignant	0.9679	Tianjin	Malignant	Malignant	Malignant	Malignant
TJ183	Malignant	0.9302	Tianjin	Malignant	Malignant	Malignant	Malignant
TJ184	Malignant	0.9889	Tianjin	Malignant	Malignant	Malignant	Malignant
TJ185	Malignant	0.9673	Tianjin	Malignant	Malignant	Malignant	Malignant
TJ186	Malignant	0.9584	Tianjin	Malignant	Malignant	Malignant	Malignant
TJ187	Malignant	0.9619	Tianjin	Malignant	Malignant	Malignant	Malignant
TJ188	Malignant	0.9451	Tianjin	Malignant	Malignant	Malignant	Malignant
TJ189	Malignant	0.9868	Tianjin	Malignant	Malignant	Malignant	Malignant
TJ190	Malignant	0.9569	Tianjin	Malignant	Malignant	Malignant	Malignant
TJ191	Malignant	0.9639	Tianjin	Malignant	Malignant	Malignant	Malignant
TJ192	Malignant	0.9622	Tianjin	Malignant	Malignant	Malignant	Malignant
TJ193	Malignant	0.9600	Tianjin	Malignant	Malignant	Malignant	Malignant
TJ194	Malignant	0.8794	Tianjin	Malignant	Malignant	Malignant	Malignant
TJ195	Malignant	0.9538	Tianjin	Malignant	Malignant	Malignant	Malignant
TJ196	Malignant	0.9755	Tianjin	Malignant	Malignant	Malignant	Malignant
TJ197	Malignant	0.8972	Tianjin	Malignant	Malignant	Benign	Malignant
TJ198	Malignant	0.9173	Tianjin	Malignant	Malignant	Malignant	Malignant
TJ199	Malignant	0.9478	Tianjin	Malignant	Malignant	Malignant	Malignant
TJ200	Malignant	0.9494	Tianjin	Malignant	Malignant	Malignant	Malignant
TJ201	Malignant	0.8735	Tianjin	Malignant	Malignant	Benign	Malignant
TJ202	Malignant	0.9750	Tianjin	Malignant	Malignant	Malignant	Malignant
TJ203	Malignant	0.9779	Tianjin	Malignant	Malignant	Benign	Malignant
TJ204	Malignant	0.8137	Tianjin	Malignant	Malignant	Malignant	Malignant
TJ205	Malignant	0.9047	Tianjin	Malignant	Malignant	Benign	Malignant
TJ206	Malignant	0.9774	Tianjin	Malignant	Malignant	Malignant	Malignant
TJ207	Malignant	0.9757	Tianjin	Malignant	Malignant	Malignant	Malignant
TJ208	Malignant	0.9520	Tianjin	Malignant	Malignant	Malignant	Malignant
TJ209	Malignant	0.9878	Tianjin	Malignant	Malignant	Malignant	Malignant
TJ210	Malignant	0.5839	Tianjin	Malignant	Malignant	Benign	Malignant
TJ211	Malignant	0.9611	Tianjin	Malignant	Malignant	Malignant	Malignant

Table S1 Continued

Patient ID	Pathological examination result	Probability predicted to be malignant by THCaDxNLP	Test set	Radiologist1 interpretation without THCaDxNLP	Radiologist2 interpretation without THCaDxNLP	Radiologist3 interpretation with THCaDxNLP	Radiologist4 interpretation with THCaDxNLP
TJ212	Malignant	0.9565	Tianjin	Malignant	Malignant	Benign	Malignant
TJ213	Malignant	0.9791	Tianjin	Malignant	Malignant	Malignant	Malignant
TJ214	Malignant	0.9668	Tianjin	Malignant	Malignant	Malignant	Malignant
TJ215	Malignant	0.9894	Tianjin	Malignant	Malignant	Malignant	Malignant
TJ216	Malignant	0.9785	Tianjin	Malignant	Malignant	Malignant	Malignant
TJ217	Malignant	0.7977	Tianjin	Malignant	Malignant	Malignant	Malignant
TJ218	Malignant	0.6411	Tianjin	Malignant	Malignant	Malignant	Malignant
TJ219	Malignant	0.6535	Tianjin	Malignant	Malignant	Malignant	Malignant
TJ220	Malignant	0.9143	Tianjin	Malignant	Malignant	Malignant	Malignant
TJ221	Malignant	0.9184	Tianjin	Malignant	Malignant	Malignant	Malignant
TJ222	Malignant	0.9215	Tianjin	Malignant	Malignant	Benign	Malignant
TJ223	Malignant	0.9586	Tianjin	Malignant	Malignant	Malignant	Malignant
TJ224	Malignant	0.9827	Tianjin	Malignant	Malignant	Malignant	Malignant
TJ225	Malignant	0.9850	Tianjin	Malignant	Malignant	Malignant	Malignant
TJ226	Malignant	0.9595	Tianjin	Malignant	Malignant	Malignant	Malignant
TJ227	Malignant	0.9865	Tianjin	Malignant	Malignant	Malignant	Malignant
TJ228	Malignant	0.9263	Tianjin	Malignant	Malignant	Malignant	Malignant
TJ229	Malignant	0.9526	Tianjin	Malignant	Malignant	Malignant	Malignant
TJ230	Malignant	0.9814	Tianjin	Malignant	Malignant	Malignant	Malignant
TJ231	Malignant	0.9033	Tianjin	Malignant	Malignant	Malignant	Malignant
TJ232	Malignant	0.8052	Tianjin	Malignant	Malignant	Malignant	Malignant
TJ233	Malignant	0.9786	Tianjin	Malignant	Malignant	Malignant	Malignant
TJ234	Malignant	0.1544	Tianjin	Malignant	Malignant	Malignant	Malignant
TJ235	Malignant	0.8843	Tianjin	Malignant	Malignant	Malignant	Benign
TJ236	Malignant	0.9692	Tianjin	Malignant	Malignant	Malignant	Malignant
TJ237	Malignant	0.9707	Tianjin	Malignant	Malignant	Malignant	Malignant
TJ238	Malignant	0.9534	Tianjin	Malignant	Malignant	Malignant	Malignant
TJ239	Malignant	0.9624	Tianjin	Malignant	Malignant	Malignant	Malignant
TJ240	Malignant	0.9222	Tianjin	Malignant	Malignant	Malignant	Malignant
TJ241	Malignant	0.9437	Tianjin	Malignant	Malignant	Malignant	Malignant
TJ242	Malignant	0.9786	Tianjin	Malignant	Malignant	Malignant	Malignant
TJ243	Malignant	0.0421	Tianjin	Malignant	Malignant	Malignant	Malignant
TJ244	Malignant	0.8576	Tianjin	Malignant	Malignant	Malignant	Malignant

Table S1 Continued

Patient ID	Pathological examination result	Probability predicted to be malignant by THCaDxNLP	Test set	Radiologist1 interpretation without THCaDxNLP	Radiologist2 interpretation without THCaDxNLP	Radiologist3 interpretation with THCaDxNLP	Radiologist4 interpretation with THCaDxNLP
TJ245	Malignant	0.9451	Tianjin	Malignant	Malignant	Malignant	Malignant
TJ246	Malignant	0.9673	Tianjin	Malignant	Malignant	Malignant	Malignant
TJ247	Malignant	0.9805	Tianjin	Malignant	Malignant	Malignant	Malignant
TJ248	Malignant	0.9464	Tianjin	Malignant	Malignant	Malignant	Malignant
TJ249	Malignant	0.9731	Tianjin	Malignant	Malignant	Malignant	Malignant
TJ250	Malignant	0.8621	Tianjin	Malignant	Malignant	Malignant	Malignant
TJ251	Malignant	0.9524	Tianjin	Malignant	Malignant	Malignant	Malignant
TJ252	Malignant	0.9415	Tianjin	Malignant	Malignant	Malignant	Malignant
TJ253	Malignant	0.9574	Tianjin	Malignant	Malignant	Malignant	Malignant
TJ254	Malignant	0.9635	Tianjin	Malignant	Malignant	Malignant	Malignant
TJ255	Malignant	0.9726	Tianjin	Malignant	Malignant	Malignant	Malignant
TJ256	Malignant	0.7288	Tianjin	Malignant	Malignant	Malignant	Malignant
TJ257	Malignant	0.8417	Tianjin	Malignant	Malignant	Malignant	Malignant
TJ258	Malignant	0.4963	Tianjin	Malignant	Malignant	Malignant	Malignant
TJ259	Malignant	0.9753	Tianjin	Malignant	Malignant	Malignant	Malignant
TJ260	Malignant	0.9198	Tianjin	Malignant	Malignant	Malignant	Malignant
TJ261	Malignant	0.9196	Tianjin	Malignant	Malignant	Malignant	Malignant
TJ262	Malignant	0.9656	Tianjin	Malignant	Malignant	Malignant	Malignant
TJ263	Malignant	0.9656	Tianjin	Malignant	Malignant	Malignant	Malignant
TJ264	Malignant	0.9827	Tianjin	Malignant	Malignant	Malignant	Malignant
TJ265	Malignant	0.9629	Tianjin	Malignant	Malignant	Malignant	Malignant
TJ266	Malignant	0.9730	Tianjin	Malignant	Malignant	Malignant	Malignant
TJ267	Malignant	0.9880	Tianjin	Malignant	Malignant	Malignant	Malignant
TJ268	Malignant	0.8742	Tianjin	Malignant	Malignant	Malignant	Malignant
TJ269	Malignant	0.9582	Tianjin	Malignant	Malignant	Malignant	Malignant
TJ270	Malignant	0.9424	Tianjin	Malignant	Malignant	Malignant	Malignant
TJ271	Malignant	0.9858	Tianjin	Malignant	Malignant	Malignant	Malignant
TJ272	Malignant	0.9735	Tianjin	Malignant	Malignant	Malignant	Malignant
TJ273	Malignant	0.9091	Tianjin	Malignant	Malignant	Malignant	Malignant
TJ274	Malignant	0.9829	Tianjin	Malignant	Malignant	Malignant	Malignant
TJ275	Malignant	0.9605	Tianjin	Malignant	Malignant	Malignant	Malignant
TJ276	Malignant	0.9840	Tianjin	Malignant	Malignant	Malignant	Malignant
TJ277	Malignant	0.9436	Tianjin	Malignant	Malignant	Malignant	Malignant

Table S1 Continued

Patient ID	Pathological examination result	Probability predicted to be malignant by THCaDxNLP	Test set	Radiologist1 interpretation without THCaDxNLP	Radiologist2 interpretation without THCaDxNLP	Radiologist3 interpretation with THCaDxNLP	Radiologist4 interpretation with THCaDxNLP
TJ278	Malignant	0.9629	Tianjin	Malignant	Malignant	Malignant	Malignant
TJ279	Malignant	0.9365	Tianjin	Malignant	Malignant	Malignant	Malignant
TJ280	Malignant	0.9820	Tianjin	Malignant	Malignant	Malignant	Malignant
TJ281	Malignant	0.9292	Tianjin	Malignant	Malignant	Malignant	Malignant
TJ282	Malignant	0.9871	Tianjin	Malignant	Malignant	Malignant	Malignant
TJ283	Malignant	0.9653	Tianjin	Malignant	Malignant	Malignant	Malignant
TJ284	Malignant	0.9585	Tianjin	Malignant	Malignant	Malignant	Malignant
TJ285	Malignant	0.9594	Tianjin	Malignant	Malignant	Malignant	Malignant
TJ286	Malignant	0.9723	Tianjin	Malignant	Malignant	Malignant	Malignant
TJ287	Malignant	0.9845	Tianjin	Malignant	Malignant	Malignant	Malignant
TJ288	Malignant	0.9536	Tianjin	Malignant	Malignant	Malignant	Malignant
TJ289	Malignant	0.9820	Tianjin	Malignant	Malignant	Malignant	Malignant
TJ290	Malignant	0.9685	Tianjin	Malignant	Malignant	Malignant	Malignant
TJ291	Malignant	0.9377	Tianjin	Malignant	Malignant	Malignant	Malignant
TJ292	Malignant	0.9800	Tianjin	Malignant	Malignant	Malignant	Malignant
TJ293	Malignant	0.9828	Tianjin	Malignant	Malignant	Malignant	Malignant
TJ294	Malignant	0.9356	Tianjin	Malignant	Malignant	Malignant	Malignant
TJ295	Malignant	0.9907	Tianjin	Malignant	Malignant	Malignant	Malignant
TJ296	Malignant	0.6577	Tianjin	Malignant	Malignant	Malignant	Malignant
TJ297	Malignant	0.9613	Tianjin	Malignant	Malignant	Malignant	Malignant
TJ298	Malignant	0.9864	Tianjin	Malignant	Malignant	Malignant	Malignant
TJ299	Malignant	0.9759	Tianjin	Malignant	Malignant	Malignant	Malignant
TJ300	Malignant	0.9793	Tianjin	Malignant	Malignant	Malignant	Malignant
TJ301	Malignant	0.9568	Tianjin	Malignant	Malignant	Malignant	Malignant
TJ302	Malignant	0.9538	Tianjin	Malignant	Malignant	Malignant	Malignant
TJ303	Malignant	0.9801	Tianjin	Malignant	Malignant	Malignant	Malignant
TJ304	Malignant	0.1024	Tianjin	Malignant	Malignant	Benign	Malignant
TJ305	Malignant	0.9634	Tianjin	Malignant	Malignant	Malignant	Malignant
TJ306	Malignant	0.9612	Tianjin	Malignant	Malignant	Malignant	Malignant
TJ307	Malignant	0.9887	Tianjin	Malignant	Malignant	Malignant	Malignant
TJ308	Malignant	0.9845	Tianjin	Malignant	Malignant	Malignant	Malignant
TJ309	Malignant	0.9390	Tianjin	Malignant	Malignant	Benign	Malignant
TJ310	Malignant	0.9294	Tianjin	Malignant	Malignant	Malignant	Malignant

Table S1 Continued

Patient ID	Pathological examination result	Probability predicted to be malignant by THCaDxNLP	Test set	Radiologist1 interpretation without THCaDxNLP	Radiologist2 interpretation without THCaDxNLP	Radiologist3 interpretation with THCaDxNLP	Radiologist4 interpretation with THCaDxNLP
TJ311	Malignant	0.9246	Tianjin	Malignant	Malignant	Malignant	Malignant
TJ312	Malignant	0.9552	Tianjin	Malignant	Malignant	Malignant	Malignant
TJ313	Malignant	0.7573	Tianjin	Malignant	Malignant	Malignant	Malignant
TJ314	Malignant	0.9776	Tianjin	Malignant	Malignant	Malignant	Malignant
TJ315	Malignant	0.9658	Tianjin	Malignant	Malignant	Malignant	Malignant
TJ316	Malignant	0.3609	Tianjin	Malignant	Malignant	Benign	Malignant
TJ317	Malignant	0.8530	Tianjin	Malignant	Malignant	Malignant	Malignant
TJ318	Malignant	0.7904	Tianjin	Malignant	Malignant	Malignant	Malignant
TJ319	Malignant	0.9400	Tianjin	Malignant	Malignant	Malignant	Malignant
TJ320	Malignant	0.9483	Tianjin	Malignant	Malignant	Malignant	Malignant
TJ321	Malignant	0.4100	Tianjin	Malignant	Malignant	Malignant	Malignant
TJ322	Malignant	0.9531	Tianjin	Malignant	Malignant	Malignant	Malignant
TJ323	Malignant	0.4024	Tianjin	Malignant	Malignant	Malignant	Malignant
TJ324	Malignant	0.9490	Tianjin	Malignant	Malignant	Malignant	Malignant
TJ325	Malignant	0.9489	Tianjin	Malignant	Malignant	Malignant	Malignant
TJ326	Malignant	0.0069	Tianjin	Malignant	Malignant	Malignant	Malignant
TJ327	Malignant	0.9696	Tianjin	Malignant	Malignant	Malignant	Malignant
TJ328	Malignant	0.8251	Tianjin	Malignant	Malignant	Malignant	Malignant
TJ329	Malignant	0.9703	Tianjin	Malignant	Malignant	Malignant	Malignant
TJ330	Malignant	0.9725	Tianjin	Malignant	Malignant	Malignant	Malignant
TJ331	Malignant	0.9811	Tianjin	Malignant	Malignant	Malignant	Malignant
TJ332	Malignant	0.9584	Tianjin	Malignant	Malignant	Malignant	Malignant
TJ333	Malignant	0.0244	Tianjin	Malignant	Malignant	Benign	Malignant
TJ334	Malignant	0.4893	Tianjin	Malignant	Malignant	Malignant	Malignant
TJ335	Malignant	0.8837	Tianjin	Malignant	Malignant	Malignant	Malignant
TJ336	Malignant	0.9817	Tianjin	Malignant	Malignant	Malignant	Malignant
TJ337	Malignant	0.3730	Tianjin	Malignant	Malignant	Benign	Malignant
TJ338	Malignant	0.9551	Tianjin	Malignant	Malignant	Malignant	Malignant
TJ339	Malignant	0.9644	Tianjin	Malignant	Malignant	Malignant	Malignant
TJ340	Malignant	0.9862	Tianjin	Malignant	Malignant	Malignant	Malignant
TJ341	Malignant	0.1439	Tianjin	Malignant	Malignant	Malignant	Malignant
TJ342	Malignant	0.9857	Tianjin	Malignant	Malignant	Malignant	Malignant
TJ343	Malignant	0.9823	Tianjin	Malignant	Malignant	Malignant	Malignant

Table S1 Continued

Patient ID	Pathological examination result	Probability predicted to be malignant by THCaDxNLP	Test set	Radiologist1 interpretation without THCaDxNLP	Radiologist2 interpretation without THCaDxNLP	Radiologist3 interpretation with THCaDxNLP	Radiologist4 interpretation with THCaDxNLP
TJ344	Malignant	0.9868	Tianjin	Malignant	Malignant	Malignant	Malignant
TJ345	Malignant	0.9137	Tianjin	Malignant	Malignant	Malignant	Malignant
TJ346	Malignant	0.9243	Tianjin	Malignant	Malignant	Malignant	Malignant
TJ347	Malignant	0.9692	Tianjin	Malignant	Malignant	Malignant	Malignant
TJ348	Malignant	0.9495	Tianjin	Malignant	Malignant	Malignant	Malignant
TJ349	Malignant	0.9790	Tianjin	Malignant	Malignant	Malignant	Malignant
TJ350	Malignant	0.9246	Tianjin	Malignant	Malignant	Malignant	Malignant
TJ351	Malignant	0.0327	Tianjin	Malignant	Malignant	Malignant	Malignant
TJ352	Malignant	0.9258	Tianjin	Malignant	Malignant	Malignant	Malignant
TJ353	Malignant	0.9777	Tianjin	Malignant	Malignant	Malignant	Malignant
TJ354	Malignant	0.9585	Tianjin	Malignant	Malignant	Malignant	Malignant
TJ355	Malignant	0.8103	Tianjin	Malignant	Malignant	Malignant	Malignant
TJ356	Malignant	0.9637	Tianjin	Malignant	Malignant	Malignant	Malignant
TJ357	Malignant	0.7031	Tianjin	Malignant	Malignant	Malignant	Malignant
TJ358	Malignant	0.9380	Tianjin	Malignant	Malignant	Malignant	Malignant
TJ359	Malignant	0.8945	Tianjin	Malignant	Malignant	Malignant	Malignant
TJ360	Malignant	0.9565	Tianjin	Malignant	Malignant	Malignant	Malignant
TJ361	Malignant	0.9704	Tianjin	Malignant	Malignant	Malignant	Malignant
TJ362	Malignant	0.0526	Tianjin	Malignant	Malignant	Malignant	Malignant
TJ363	Malignant	0.0397	Tianjin	Malignant	Malignant	Malignant	Malignant
TJ364	Malignant	0.9850	Tianjin	Malignant	Malignant	Malignant	Malignant
TJ365	Malignant	0.9716	Tianjin	Malignant	Malignant	Malignant	Malignant
TJ366	Malignant	0.9601	Tianjin	Malignant	Malignant	Malignant	Malignant
TJ367	Malignant	0.9690	Tianjin	Malignant	Malignant	Malignant	Malignant
TJ368	Malignant	0.9282	Tianjin	Malignant	Malignant	Malignant	Malignant
TJ369	Malignant	0.9593	Tianjin	Malignant	Malignant	Malignant	Malignant
TJ370	Malignant	0.9495	Tianjin	Malignant	Malignant	Malignant	Malignant
TJ371	Malignant	0.6981	Tianjin	Malignant	Malignant	Malignant	Malignant
TJ372	Malignant	0.9589	Tianjin	Malignant	Malignant	Malignant	Malignant
TJ373	Malignant	0.9499	Tianjin	Malignant	Malignant	Malignant	Malignant
TJ374	Malignant	0.0076	Tianjin	Malignant	Malignant	Benign	Malignant
TJ375	Malignant	0.9202	Tianjin	Malignant	Malignant	Malignant	Malignant
TJ376	Malignant	0.9667	Tianjin	Malignant	Malignant	Malignant	Malignant

Table S1 Continued

Patient ID	Pathological examination result	Probability predicted to be malignant by THCaDxNLP	Test set	Radiologist1 interpretation without THCaDxNLP	Radiologist2 interpretation without THCaDxNLP	Radiologist3 interpretation with THCaDxNLP	Radiologist4 interpretation with THCaDxNLP
TJ377	Malignant	0.9773	Tianjin	Malignant	Malignant	Malignant	Malignant
TJ378	Malignant	0.9504	Tianjin	Malignant	Malignant	Malignant	Malignant
TJ379	Malignant	0.9692	Tianjin	Malignant	Malignant	Benign	Malignant
TJ380	Malignant	0.9552	Tianjin	Malignant	Malignant	Malignant	Malignant
TJ381	Malignant	0.9798	Tianjin	Malignant	Malignant	Malignant	Malignant
TJ382	Malignant	0.9076	Tianjin	Malignant	Malignant	Malignant	Malignant
TJ383	Malignant	0.8524	Tianjin	Malignant	Malignant	Benign	Malignant
TJ384	Malignant	0.9703	Tianjin	Malignant	Malignant	Malignant	Malignant
TJ385	Malignant	0.3925	Tianjin	Malignant	Malignant	Malignant	Malignant
TJ386	Malignant	0.9665	Tianjin	Malignant	Malignant	Malignant	Malignant
TJ387	Malignant	0.9846	Tianjin	Malignant	Malignant	Malignant	Malignant
TJ388	Malignant	0.9894	Tianjin	Malignant	Malignant	Malignant	Malignant
TJ389	Malignant	0.9436	Tianjin	Malignant	Malignant	Malignant	Malignant
TJ390	Malignant	0.0093	Tianjin	Malignant	Malignant	Benign	Malignant
TJ391	Malignant	0.9734	Tianjin	Malignant	Malignant	Malignant	Malignant
TJ392	Malignant	0.9782	Tianjin	Malignant	Malignant	Malignant	Malignant
TJ393	Malignant	0.9805	Tianjin	Malignant	Malignant	Malignant	Malignant
TJ394	Malignant	0.9862	Tianjin	Malignant	Malignant	Malignant	Malignant
TJ395	Malignant	0.9640	Tianjin	Malignant	Malignant	Malignant	Malignant
TJ396	Malignant	0.9579	Tianjin	Malignant	Malignant	Malignant	Malignant
TJ397	Malignant	0.9180	Tianjin	Malignant	Malignant	Malignant	Malignant
TJ398	Malignant	0.8964	Tianjin	Malignant	Malignant	Malignant	Malignant
TJ399	Malignant	0.8923	Tianjin	Malignant	Malignant	Benign	Malignant
TJ400	Malignant	0.9617	Tianjin	Malignant	Malignant	Malignant	Malignant
TJ401	Malignant	0.9885	Tianjin	Malignant	Malignant	Malignant	Malignant
TJ402	Malignant	0.9657	Tianjin	Malignant	Malignant	Malignant	Malignant
TJ403	Malignant	0.9259	Tianjin	Malignant	Malignant	Malignant	Malignant
TJ404	Malignant	0.8826	Tianjin	Malignant	Malignant	Malignant	Malignant
TJ405	Malignant	0.9812	Tianjin	Malignant	Malignant	Malignant	Malignant
TJ406	Malignant	0.8890	Tianjin	Malignant	Malignant	Malignant	Malignant
TJ407	Malignant	0.9867	Tianjin	Malignant	Malignant	Malignant	Malignant
TJ408	Malignant	0.9468	Tianjin	Malignant	Malignant	Malignant	Malignant
TJ409	Malignant	0.9461	Tianjin	Malignant	Malignant	Malignant	Malignant

Table S1 Continued

Patient ID	Pathological examination result	Probability predicted to be malignant by THCaDxNLP	Test set	Radiologist1 interpretation without THCaDxNLP	Radiologist2 interpretation without THCaDxNLP	Radiologist3 interpretation with THCaDxNLP	Radiologist4 interpretation with THCaDxNLP
TJ410	Malignant	0.9791	Tianjin	Malignant	Malignant	Malignant	Malignant
TJ411	Malignant	0.9886	Tianjin	Malignant	Malignant	Malignant	Malignant
TJ412	Malignant	0.9823	Tianjin	Malignant	Malignant	Malignant	Malignant
TJ413	Malignant	0.9140	Tianjin	Malignant	Malignant	Malignant	Malignant
TJ414	Malignant	0.9200	Tianjin	Malignant	Malignant	Malignant	Malignant
TJ415	Malignant	0.9306	Tianjin	Malignant	Malignant	Malignant	Malignant
TJ416	Malignant	0.9551	Tianjin	Malignant	Malignant	Malignant	Malignant
TJ417	Malignant	0.9671	Tianjin	Malignant	Malignant	Malignant	Malignant
TJ418	Malignant	0.8799	Tianjin	Malignant	Malignant	Malignant	Malignant
TJ419	Malignant	0.9735	Tianjin	Malignant	Malignant	Malignant	Malignant
TJ420	Malignant	0.0073	Tianjin	Malignant	Malignant	Benign	Benign
TJ421	Malignant	0.9606	Tianjin	Malignant	Malignant	Malignant	Malignant
TJ422	Malignant	0.9798	Tianjin	Malignant	Malignant	Malignant	Malignant
TJ423	Malignant	0.9185	Tianjin	Malignant	Malignant	Malignant	Malignant
TJ424	Malignant	0.4676	Tianjin	Malignant	Malignant	Benign	Malignant
TJ425	Malignant	0.9213	Tianjin	Malignant	Malignant	Malignant	Malignant
TJ426	Malignant	0.9793	Tianjin	Malignant	Malignant	Malignant	Malignant
TJ427	Malignant	0.0188	Tianjin	Malignant	Malignant	Benign	Malignant
TJ428	Malignant	0.7927	Tianjin	Malignant	Malignant	Malignant	Malignant
TJ429	Malignant	0.0483	Tianjin	Malignant	Malignant	Benign	Malignant
TJ430	Malignant	0.6454	Tianjin	Malignant	Malignant	Malignant	Malignant
TJ431	Malignant	0.9763	Tianjin	Malignant	Malignant	Malignant	Malignant
TJ432	Malignant	0.9622	Tianjin	Malignant	Malignant	Malignant	Malignant
TJ433	Malignant	0.9714	Tianjin	Malignant	Malignant	Malignant	Malignant
TJ434	Malignant	0.9765	Tianjin	Malignant	Malignant	Malignant	Malignant
TJ435	Malignant	0.9748	Tianjin	Malignant	Malignant	Malignant	Malignant
TJ436	Malignant	0.8982	Tianjin	Malignant	Malignant	Malignant	Malignant
TJ437	Malignant	0.9816	Tianjin	Malignant	Malignant	Malignant	Malignant
TJ438	Malignant	0.8810	Tianjin	Malignant	Malignant	Malignant	Malignant
TJ439	Malignant	0.9369	Tianjin	Malignant	Malignant	Benign	Malignant
WH1	Malignant	0.9455	Weihai	Malignant	Benign	Malignant	Malignant
WH2	Malignant	0.9924	Weihai	Malignant	Benign	Malignant	Malignant
WH3	Malignant	0.9898	Weihai	Malignant	Malignant	Malignant	Malignant

Table S1 Continued

Patient ID	Pathological examination result	Probability predicted to be malignant by THCaDxNLP	Test set	Radiologist1 interpretation without THCaDxNLP	Radiologist2 interpretation without THCaDxNLP	Radiologist3 interpretation with THCaDxNLP	Radiologist4 interpretation with THCaDxNLP
WH4	Benign	0.0025	Weihai	Benign	Benign	Benign	Benign
WH5	Malignant	0.9883	Weihai	Malignant	Malignant	Malignant	Malignant
WH6	Benign	0.0025	Weihai	Benign	Benign	Benign	Benign
WH7	Malignant	0.9841	Weihai	Malignant	Malignant	Malignant	Malignant
WH8	Malignant	0.9934	Weihai	Malignant	Malignant	Malignant	Malignant
WH9	Benign	0.0014	Weihai	Benign	Benign	Benign	Benign
WH10	Malignant	0.9883	Weihai	Malignant	Malignant	Malignant	Malignant
WH11	Malignant	0.0901	Weihai	Malignant	Malignant	Malignant	Malignant
WH12	Malignant	0.7939	Weihai	Benign	Malignant	Malignant	Malignant
WH13	Benign	0.0009	Weihai	Benign	Malignant	Benign	Benign
WH14	Benign	0.0035	Weihai	Benign	Benign	Benign	Benign
WH15	Malignant	0.9923	Weihai	Malignant	Malignant	Malignant	Malignant
WH16	Malignant	0.9417	Weihai	Malignant	Malignant	Malignant	Malignant
WH17	Benign	0.0028	Weihai	Benign	Benign	Benign	Benign
WH18	Benign	0.0068	Weihai	Malignant	Benign	Benign	Benign
WH19	Malignant	0.9816	Weihai	Malignant	Benign	Malignant	Malignant
WH20	Malignant	0.0039	Weihai	Malignant	Malignant	Malignant	Malignant
WH21	Malignant	0.9923	Weihai	Malignant	Benign	Malignant	Malignant
WH22	Malignant	0.9878	Weihai	Malignant	Malignant	Malignant	Malignant
WH23	Benign	0.0041	Weihai	Benign	Benign	Benign	Malignant
WH24	Benign	0.0030	Weihai	Benign	Benign	Benign	Benign
WH25	Malignant	0.9914	Weihai	Malignant	Malignant	Malignant	Malignant
WH26	Malignant	0.7815	Weihai	Malignant	Malignant	Malignant	Malignant
WH27	Malignant	0.9940	Weihai	Malignant	Malignant	Malignant	Malignant
WH28	Malignant	0.9596	Weihai	Malignant	Malignant	Malignant	Malignant
WH29	Benign	0.0021	Weihai	Malignant	Benign	Benign	Benign
WH30	Benign	0.0150	Weihai	Benign	Benign	Benign	Benign
WH31	Malignant	0.0449	Weihai	Malignant	Malignant	Malignant	Malignant
WH32	Malignant	0.7678	Weihai	Malignant	Benign	Malignant	Malignant
WH33	Malignant	0.0025	Weihai	Malignant	Benign	Malignant	Malignant
WH34	Malignant	0.9913	Weihai	Malignant	Malignant	Malignant	Malignant
WH35	Benign	0.0044	Weihai	Benign	Benign	Benign	Benign
WH36	Malignant	0.9947	Weihai	Malignant	Malignant	Malignant	Malignant

Table S1 Continued

Patient ID	Pathological examination result	Probability predicted to be malignant by THCaDxNLP	Test set	Radiologist1 interpretation without THCaDxNLP	Radiologist2 interpretation without THCaDxNLP	Radiologist3 interpretation with THCaDxNLP	Radiologist4 interpretation with THCaDxNLP
WH37	Malignant	0.9937	Weihai	Malignant	Malignant	Malignant	Malignant
WH38	Malignant	0.9818	Weihai	Malignant	Malignant	Malignant	Malignant
WH39	Malignant	0.9818	Weihai	Malignant	Malignant	Benign	Malignant
WH40	Malignant	0.6152	Weihai	Malignant	Benign	Malignant	Malignant
WH41	Malignant	0.9952	Weihai	Malignant	Malignant	Malignant	Malignant
WH42	Malignant	0.9860	Weihai	Malignant	Benign	Malignant	Malignant
WH43	Malignant	0.9918	Weihai	Malignant	Malignant	Malignant	Malignant
WH44	Malignant	0.9732	Weihai	Malignant	Malignant	Malignant	Malignant
WH45	Malignant	0.3879	Weihai	Malignant	Benign	Malignant	Malignant
WH46	Benign	0.0013	Weihai	Benign	Benign	Benign	Benign
WH47	Benign	0.0020	Weihai	Benign	Benign	Benign	Benign
WH48	Benign	0.0041	Weihai	Benign	Benign	Benign	Benign
WH49	Benign	0.0009	Weihai	Benign	Benign	Benign	Benign
WH50	Malignant	0.9908	Weihai	Malignant	Malignant	Malignant	Malignant
WH51	Malignant	0.9582	Weihai	Malignant	Malignant	Malignant	Malignant
WH52	Benign	0.2599	Weihai	Benign	Benign	Benign	Benign
WH53	Benign	0.0019	Weihai	Benign	Benign	Benign	Benign
WH54	Benign	0.0043	Weihai	Malignant	Benign	Benign	Benign
WH55	Malignant	0.9933	Weihai	Malignant	Benign	Malignant	Malignant
WH56	Benign	0.1501	Weihai	Malignant	Benign	Benign	Benign
WH57	Malignant	0.9919	Weihai	Malignant	Benign	Malignant	Malignant
WH58	Benign	0.0249	Weihai	Malignant	Benign	Malignant	Benign
WH59	Malignant	0.5846	Weihai	Malignant	Malignant	Malignant	Malignant
WH60	Malignant	0.9878	Weihai	Malignant	Benign	Malignant	Malignant
WH61	Malignant	0.2163	Weihai	Malignant	Benign	Malignant	Malignant
WH62	Malignant	0.9699	Weihai	Malignant	Malignant	Malignant	Malignant
WH63	Benign	0.0008	Weihai	Benign	Benign	Benign	Benign
WH64	Benign	0.0007	Weihai	Benign	Benign	Benign	Benign
WH65	Malignant	0.6509	Weihai	Malignant	Malignant	Malignant	Malignant
WH66	Malignant	0.4082	Weihai	Malignant	Malignant	Malignant	Malignant
WH67	Benign	0.8741	Weihai	Malignant	Benign	Benign	Benign
WH68	Benign	0.0041	Weihai	Benign	Benign	Benign	Benign
WH69	Malignant	0.9446	Weihai	Malignant	Malignant	Malignant	Malignant

Table S1 Continued

Patient ID	Pathological examination result	Probability predicted to be malignant by THCaDxNLP	Test set	Radiologist1 interpretation without THCaDxNLP	Radiologist2 interpretation without THCaDxNLP	Radiologist3 interpretation with THCaDxNLP	Radiologist4 interpretation with THCaDxNLP
WH70	Benign	0.0047	Weihai	Benign	Benign	Benign	Benign
WH71	Malignant	0.9889	Weihai	Malignant	Benign	Malignant	Malignant
WH72	Malignant	0.9920	Weihai	Malignant	Benign	Malignant	Malignant
WH73	Malignant	0.9804	Weihai	Malignant	Malignant	Malignant	Malignant
WH74	Benign	0.0009	Weihai	Benign	Malignant	Benign	Benign
WH75	Malignant	0.0220	Weihai	Malignant	Malignant	Malignant	Malignant
WH76	Benign	0.0047	Weihai	Malignant	Benign	Benign	Benign
WH77	Malignant	0.9376	Weihai	Malignant	Malignant	Malignant	Malignant
WH78	Benign	0.0066	Weihai	Benign	Benign	Benign	Benign
WH79	Benign	0.0019	Weihai	Benign	Benign	Benign	Benign
WH80	Benign	0.0039	Weihai	Benign	Benign	Benign	Benign
WH81	Benign	0.1205	Weihai	Benign	Benign	Benign	Benign
WH82	Malignant	0.9088	Weihai	Malignant	Malignant	Malignant	Malignant
WH83	Benign	0.0026	Weihai	Benign	Benign	Benign	Benign
WH84	Benign	0.0014	Weihai	Malignant	Benign	Benign	Benign
WH85	Benign	0.0031	Weihai	Benign	Benign	Benign	Benign
WH86	Benign	0.0005	Weihai	Benign	Benign	Benign	Benign
WH87	Malignant	0.9427	Weihai	Malignant	Benign	Malignant	Malignant
WH88	Malignant	0.9155	Weihai	Malignant	Malignant	Malignant	Malignant
WH89	Benign	0.9853	Weihai	Malignant	Benign	Malignant	Malignant
WH90	Malignant	0.2305	Weihai	Malignant	Malignant	Malignant	Malignant
WH91	Malignant	0.9927	Weihai	Malignant	Malignant	Malignant	Malignant
WH92	Benign	0.6068	Weihai	Benign	Benign	Benign	Benign
WH93	Malignant	0.0013	Weihai	Benign	Benign	Benign	Malignant
WH94	Malignant	0.9937	Weihai	Malignant	Malignant	Malignant	Malignant
WH95	Benign	0.0101	Weihai	Benign	Benign	Benign	Benign
WH96	Malignant	0.9856	Weihai	Malignant	Malignant	Malignant	Malignant
WH97	Benign	0.7811	Weihai	Malignant	Benign	Malignant	Malignant
WH98	Malignant	0.9942	Weihai	Malignant	Malignant	Malignant	Malignant
WH99	Malignant	0.9946	Weihai	Malignant	Malignant	Malignant	Malignant
WH100	Malignant	0.9912	Weihai	Malignant	Malignant	Malignant	Malignant
WH101	Malignant	0.9899	Weihai	Malignant	Malignant	Malignant	Malignant
WH102	Benign	0.0084	Weihai	Benign	Malignant	Benign	Malignant

Table S1 Continued

Patient ID	Pathological examination result	Probability predicted to be malignant by THCaDxNLP	Test set	Radiologist1 interpretation without THCaDxNLP	Radiologist2 interpretation without THCaDxNLP	Radiologist3 interpretation with THCaDxNLP	Radiologist4 interpretation with THCaDxNLP
WH103	Malignant	0.9933	Weihai	Malignant	Malignant	Malignant	Malignant
WH104	Benign	0.0016	Weihai	Benign	Benign	Benign	Benign
WH105	Benign	0.5697	Weihai	Benign	Benign	Benign	Benign
WH106	Malignant	0.9821	Weihai	Malignant	Malignant	Malignant	Malignant
WH107	Benign	0.0046	Weihai	Benign	Benign	Benign	Benign
WH108	Benign	0.0486	Weihai	Malignant	Benign	Benign	Benign
WH109	Malignant	0.9803	Weihai	Malignant	Malignant	Malignant	Malignant
WH110	Malignant	0.9906	Weihai	Malignant	Malignant	Malignant	Malignant
WH111	Benign	0.0043	Weihai	Benign	Benign	Benign	Benign
WH112	Benign	0.0009	Weihai	Benign	Benign	Benign	Benign
WH113	Malignant	0.6738	Weihai	Malignant	Benign	Malignant	Malignant
WH114	Benign	0.0015	Weihai	Benign	Benign	Benign	Benign
WH115	Benign	0.0042	Weihai	Benign	Benign	Benign	Benign
WH116	Malignant	0.9845	Weihai	Malignant	Benign	Malignant	Malignant
WH117	Malignant	0.9621	Weihai	Malignant	Benign	Malignant	Malignant
WH118	Benign	0.0021	Weihai	Benign	Benign	Benign	Benign
WH119	Benign	0.0485	Weihai	Benign	Benign	Benign	Benign
WH120	Benign	0.0012	Weihai	Benign	Benign	Benign	Benign
WH121	Benign	0.5307	Weihai	Malignant	Benign	Benign	Benign
WH122	Benign	0.5896	Weihai	Benign	Benign	Benign	Benign
WH123	Malignant	0.9946	Weihai	Malignant	Malignant	Malignant	Malignant
WH124	Benign	0.0037	Weihai	Benign	Benign	Benign	Benign
WH125	Malignant	0.9932	Weihai	Malignant	Malignant	Malignant	Malignant
WH126	Benign	0.0202	Weihai	Benign	Benign	Benign	Benign
WH127	Benign	0.0056	Weihai	Benign	Benign	Benign	Benign
WH128	Benign	0.0015	Weihai	Benign	Benign	Benign	Benign
WH129	Benign	0.9722	Weihai	Benign	Benign	Benign	Benign
WH130	Benign	0.4291	Weihai	Benign	Benign	Benign	Benign
WH131	Benign	0.2476	Weihai	Benign	Benign	Benign	Benign
WH132	Malignant	0.7376	Weihai	Malignant	Benign	Malignant	Malignant
WH133	Malignant	0.9469	Weihai	Malignant	Benign	Malignant	Malignant
WH134	Benign	0.1710	Weihai	Malignant	Benign	Benign	Benign
WH135	Malignant	0.9589	Weihai	Malignant	Benign	Malignant	Malignant

Table S1 Continued

Patient ID	Pathological examination result	Probability predicted to be malignant by THCaDxNLP	Test set	Radiologist1 interpretation without THCaDxNLP	Radiologist2 interpretation without THCaDxNLP	Radiologist3 interpretation with THCaDxNLP	Radiologist4 interpretation with THCaDxNLP
WH136	Malignant	0.9940	Weihai	Malignant	Malignant	Malignant	Malignant
WH137	Malignant	0.5010	Weihai	Malignant	Benign	Malignant	Malignant
WH138	Malignant	0.8948	Weihai	Malignant	Benign	Malignant	Malignant
WH139	Malignant	0.9756	Weihai	Malignant	Benign	Malignant	Malignant
WH140	Benign	0.0012	Weihai	Benign	Benign	Benign	Benign
WH141	Benign	0.2524	Weihai	Malignant	Benign	Benign	Benign
WH142	Malignant	0.9888	Weihai	Malignant	Benign	Malignant	Malignant
WH143	Malignant	0.7765	Weihai	Malignant	Benign	Malignant	Malignant
WH144	Malignant	0.9929	Weihai	Malignant	Malignant	Malignant	Malignant
WH145	Benign	0.0011	Weihai	Benign	Benign	Benign	Benign
WH146	Malignant	0.9932	Weihai	Malignant	Malignant	Malignant	Malignant
WH147	Benign	0.0095	Weihai	Benign	Benign	Benign	Benign
WH148	Malignant	0.3146	Weihai	Malignant	Benign	Malignant	Malignant
WH149	Malignant	0.9927	Weihai	Malignant	Malignant	Malignant	Malignant
WH150	Benign	0.0300	Weihai	Benign	Benign	Benign	Malignant
WH151	Malignant	0.9894	Weihai	Malignant	Benign	Malignant	Malignant
WH152	Malignant	0.9928	Weihai	Malignant	Malignant	Malignant	Malignant
WH153	Malignant	0.1940	Weihai	Malignant	Benign	Malignant	Malignant
WH154	Benign	0.0376	Weihai	Benign	Benign	Benign	Benign
WH155	Benign	0.0013	Weihai	Benign	Benign	Benign	Benign
WH156	Malignant	0.0057	Weihai	Malignant	Benign	Malignant	Malignant
WH157	Benign	0.0006	Weihai	Benign	Benign	Benign	Benign
WH158	Benign	0.9766	Weihai	Benign	Benign	Benign	Benign
WH159	Malignant	0.3909	Weihai	Benign	Malignant	Malignant	Malignant
WH160	Benign	0.0065	Weihai	Benign	Benign	Benign	Benign
WH161	Benign	0.1984	Weihai	Malignant	Benign	Malignant	Benign
WH162	Malignant	0.9759	Weihai	Malignant	Malignant	Malignant	Malignant
WH163	Benign	0.4337	Weihai	Malignant	Benign	Benign	Benign
WH164	Benign	0.0019	Weihai	Benign	Benign	Benign	Benign
WH165	Benign	0.0006	Weihai	Benign	Benign	Benign	Benign
WH166	Benign	0.0012	Weihai	Benign	Benign	Benign	Benign
WH167	Benign	0.9305	Weihai	Malignant	Benign	Benign	Malignant
WH168	Malignant	0.6547	Weihai	Malignant	Benign	Malignant	Malignant

Table S1 Continued

Patient ID	Pathological examination result	Probability predicted to be malignant by THCaDxNLP	Test set	Radiologist1 interpretation without THCaDxNLP	Radiologist2 interpretation without THCaDxNLP	Radiologist3 interpretation with THCaDxNLP	Radiologist4 interpretation with THCaDxNLP
WH169	Benign	0.0008	Weihai	Benign	Benign	Benign	Benign
WH170	Benign	0.0185	Weihai	Benign	Benign	Benign	Benign
WH171	Benign	0.0013	Weihai	Benign	Benign	Benign	Benign
WH172	Benign	0.0013	Weihai	Malignant	Benign	Benign	Benign
WH173	Benign	0.0036	Weihai	Benign	Benign	Benign	Benign
WH174	Benign	0.0067	Weihai	Benign	Benign	Benign	Benign
WH175	Malignant	0.0128	Weihai	Malignant	Benign	Malignant	Malignant
WH176	Malignant	0.9901	Weihai	Malignant	Benign	Malignant	Malignant
WH177	Malignant	0.9854	Weihai	Malignant	Malignant	Malignant	Malignant
WH178	Benign	0.0129	Weihai	Benign	Malignant	Benign	Benign
WH179	Malignant	0.7397	Weihai	Malignant	Malignant	Malignant	Malignant
WH180	Malignant	0.9928	Weihai	Malignant	Malignant	Malignant	Malignant
WH181	Malignant	0.9541	Weihai	Malignant	Malignant	Malignant	Malignant
WH182	Benign	0.0016	Weihai	Benign	Benign	Benign	Benign
WH183	Benign	0.0011	Weihai	Benign	Benign	Benign	Benign
WH184	Malignant	0.9767	Weihai	Malignant	Benign	Malignant	Malignant
WH185	Benign	0.0011	Weihai	Benign	Benign	Benign	Benign
WH186	Benign	0.0101	Weihai	Benign	Malignant	Benign	Benign
WH187	Malignant	0.7900	Weihai	Malignant	Malignant	Malignant	Malignant
WH188	Malignant	0.3340	Weihai	Malignant	Malignant	Malignant	Malignant
WH189	Benign	0.0024	Weihai	Benign	Benign	Benign	Benign
WH190	Malignant	0.9921	Weihai	Malignant	Malignant	Malignant	Malignant
WH191	Benign	0.0058	Weihai	Benign	Benign	Benign	Benign
WH192	Benign	0.0018	Weihai	Benign	Benign	Benign	Benign
WH193	Benign	0.1656	Weihai	Benign	Benign	Benign	Benign
WH194	Malignant	0.5957	Weihai	Benign	Benign	Malignant	Malignant
WH195	Malignant	0.9763	Weihai	Malignant	Benign	Malignant	Malignant
WH196	Benign	0.3453	Weihai	Benign	Benign	Benign	Benign
WH197	Malignant	0.6753	Weihai	Malignant	Benign	Malignant	Malignant
WH198	Benign	0.0109	Weihai	Benign	Benign	Benign	Benign
WH199	Malignant	0.9931	Weihai	Malignant	Malignant	Malignant	Malignant
WH200	Benign	0.0155	Weihai	Benign	Benign	Benign	Benign
WH201	Benign	0.0009	Weihai	Benign	Benign	Benign	Benign

Table S1 Continued

Patient ID	Pathological examination result	Probability predicted to be malignant by THCaDxNLP	Test set	Radiologist1 interpretation without THCaDxNLP	Radiologist2 interpretation without THCaDxNLP	Radiologist3 interpretation with THCaDxNLP	Radiologist4 interpretation with THCaDxNLP
WH202	Benign	0.0058	Weihai	Benign	Benign	Benign	Benign
WH203	Malignant	0.9863	Weihai	Malignant	Malignant	Malignant	Malignant
WH204	Malignant	0.9832	Weihai	Malignant	Malignant	Malignant	Malignant
WH205	Benign	0.0010	Weihai	Benign	Benign	Benign	Benign
WH206	Malignant	0.9936	Weihai	Malignant	Malignant	Malignant	Malignant
WH207	Malignant	0.9915	Weihai	Malignant	Benign	Malignant	Malignant
WH208	Benign	0.9368	Weihai	Malignant	Benign	Benign	Benign
WH209	Benign	0.0109	Weihai	Benign	Benign	Benign	Benign
WH210	Benign	0.0009	Weihai	Benign	Benign	Benign	Benign
WH211	Malignant	0.9933	Weihai	Malignant	Benign	Malignant	Malignant
WH212	Benign	0.1984	Weihai	Benign	Benign	Benign	Benign
WH213	Malignant	0.0016	Weihai	Malignant	Benign	Malignant	Malignant
WH214	Benign	0.0011	Weihai	Benign	Benign	Benign	Benign
WH215	Benign	0.0026	Weihai	Benign	Benign	Benign	Benign
WH216	Benign	0.0009	Weihai	Benign	Benign	Benign	Benign
WH217	Benign	0.9861	Weihai	Benign	Benign	Benign	Benign
WH218	Malignant	0.9892	Weihai	Malignant	Benign	Malignant	Malignant
WH219	Benign	0.0191	Weihai	Benign	Benign	Benign	Benign
WH220	Benign	0.0159	Weihai	Benign	Benign	Malignant	Malignant
WH221	Benign	0.0178	Weihai	Benign	Benign	Benign	Benign
WH222	Malignant	0.2255	Weihai	Malignant	Benign	Malignant	Malignant
WH223	Malignant	0.9035	Weihai	Malignant	Benign	Malignant	Malignant
WH224	Benign	0.0933	Weihai	Benign	Benign	Benign	Benign
WH225	Benign	0.0013	Weihai	Benign	Benign	Benign	Benign
WH226	Benign	0.6719	Weihai	Benign	Benign	Benign	Benign
WH227	Benign	0.1005	Weihai	Benign	Benign	Benign	Benign
WH228	Benign	0.1475	Weihai	Benign	Benign	Benign	Benign
WH229	Benign	0.9243	Weihai	Benign	Benign	Benign	Benign
WH230	Malignant	0.9912	Weihai	Malignant	Benign	Malignant	Malignant
WH231	Benign	0.0009	Weihai	Benign	Benign	Benign	Benign
WH232	Malignant	0.9745	Weihai	Malignant	Benign	Malignant	Malignant
WH233	Malignant	0.9880	Weihai	Malignant	Malignant	Malignant	Malignant
WH234	Benign	0.0509	Weihai	Benign	Benign	Benign	Benign

Table S1 Continued

Patient ID	Pathological examination result	Probability predicted to be malignant by THCaDxNLP	Test set	Radiologist1 interpretation without THCaDxNLP	Radiologist2 interpretation without THCaDxNLP	Radiologist3 interpretation with THCaDxNLP	Radiologist4 interpretation with THCaDxNLP
WH235	Malignant	0.9926	Weihai	Malignant	Malignant	Malignant	Malignant
WH236	Benign	0.8339	Weihai	Benign	Benign	Malignant	Benign
WH237	Benign	0.0021	Weihai	Benign	Benign	Benign	Benign
WH238	Benign	0.1321	Weihai	Benign	Benign	Benign	Benign
WH239	Benign	0.0008	Weihai	Benign	Benign	Benign	Benign
WH240	Malignant	0.9807	Weihai	Malignant	Benign	Malignant	Malignant
WH241	Malignant	0.9344	Weihai	Malignant	Malignant	Malignant	Malignant
WH242	Benign	0.0016	Weihai	Benign	Benign	Benign	Benign
WH243	Benign	0.0010	Weihai	Benign	Benign	Benign	Benign
WH244	Benign	0.0018	Weihai	Benign	Benign	Benign	Benign
WH245	Benign	0.0011	Weihai	Benign	Benign	Benign	Benign
WH246	Benign	0.2033	Weihai	Benign	Benign	Malignant	Malignant
WH247	Benign	0.0030	Weihai	Benign	Benign	Benign	Benign
WH248	Benign	0.1841	Weihai	Benign	Benign	Benign	Benign
WH249	Malignant	0.9915	Weihai	Malignant	Benign	Malignant	Malignant
WH250	Benign	0.0205	Weihai	Benign	Benign	Benign	Benign
WH251	Benign	0.0806	Weihai	Benign	Benign	Benign	Benign
WH252	Malignant	0.7751	Weihai	Malignant	Malignant	Malignant	Malignant
WH253	Malignant	0.9882	Weihai	Malignant	Malignant	Malignant	Malignant
WH254	Malignant	0.1910	Weihai	Malignant	Benign	Benign	Malignant
WH255	Benign	0.0291	Weihai	Benign	Benign	Benign	Benign
WH256	Benign	0.9830	Weihai	Benign	Benign	Malignant	Malignant
WH257	Benign	0.0011	Weihai	Benign	Benign	Benign	Benign
WH258	Malignant	0.6713	Weihai	Malignant	Malignant	Malignant	Malignant
WH259	Malignant	0.9925	Weihai	Malignant	Malignant	Malignant	Malignant
WH260	Malignant	0.9938	Weihai	Benign	Benign	Malignant	Malignant
WH261	Malignant	0.7875	Weihai	Malignant	Malignant	Malignant	Malignant
WH262	Benign	0.0072	Weihai	Benign	Benign	Benign	Benign
WH263	Benign	0.0023	Weihai	Benign	Benign	Benign	Benign
WH264	Malignant	0.9259	Weihai	Benign	Malignant	Benign	Malignant
WH265	Benign	0.0015	Weihai	Benign	Benign	Benign	Benign
WH266	Malignant	0.9867	Weihai	Malignant	Malignant	Malignant	Malignant
WH267	Malignant	0.9846	Weihai	Malignant	Benign	Malignant	Malignant

Table S1 Continued

Patient ID	Pathological examination result	Probability predicted to be malignant by THCaDxNLP	Test set	Radiologist1 interpretation without THCaDxNLP	Radiologist2 interpretation without THCaDxNLP	Radiologist3 interpretation with THCaDxNLP	Radiologist4 interpretation with THCaDxNLP
WH268	Benign	0.0028	Weihai	Benign	Benign	Benign	Benign
WH269	Benign	0.0086	Weihai	Benign	Benign	Benign	Benign
WH270	Malignant	0.9612	Weihai	Malignant	Benign	Malignant	Malignant
WH271	Malignant	0.0190	Weihai	Malignant	Malignant	Benign	Benign
WH272	Malignant	0.0208	Weihai	Malignant	Benign	Malignant	Malignant
WH273	Benign	0.0010	Weihai	Benign	Benign	Benign	Benign
WH274	Benign	0.0015	Weihai	Benign	Benign	Benign	Benign
WH275	Malignant	0.8230	Weihai	Malignant	Benign	Malignant	Malignant
WH276	Benign	0.0079	Weihai	Benign	Benign	Benign	Benign
WH277	Benign	0.0760	Weihai	Benign	Benign	Malignant	Benign
WH278	Benign	0.0015	Weihai	Benign	Benign	Benign	Benign
WH279	Malignant	0.9933	Weihai	Malignant	Malignant	Malignant	Malignant
WH280	Malignant	0.9894	Weihai	Malignant	Benign	Malignant	Malignant
WH281	Benign	0.0012	Weihai	Benign	Malignant	Benign	Benign
WH282	Malignant	0.9303	Weihai	Malignant	Malignant	Malignant	Malignant
WH283	Benign	0.0448	Weihai	Benign	Benign	Benign	Benign
WH284	Benign	0.0025	Weihai	Benign	Benign	Benign	Benign
WH285	Malignant	0.0104	Weihai	Malignant	Benign	Malignant	Malignant
WH286	Benign	0.0014	Weihai	Benign	Benign	Benign	Benign
WH287	Benign	0.4062	Weihai	Benign	Benign	Benign	Benign
WH288	Benign	0.0012	Weihai	Benign	Benign	Benign	Benign
WH289	Malignant	0.9882	Weihai	Malignant	Malignant	Malignant	Malignant
WH290	Benign	0.0012	Weihai	Benign	Benign	Benign	Benign
WH291	Benign	0.0023	Weihai	Benign	Benign	Benign	Benign
WH292	Malignant	0.9759	Weihai	Malignant	Malignant	Malignant	Malignant
WH293	Benign	0.0040	Weihai	Benign	Benign	Malignant	Benign
WH294	Malignant	0.9902	Weihai	Malignant	Benign	Malignant	Malignant
WH295	Malignant	0.9919	Weihai	Malignant	Malignant	Malignant	Malignant
WH296	Benign	0.9736	Weihai	Benign	Benign	Malignant	Malignant
WH297	Malignant	0.3398	Weihai	Malignant	Malignant	Malignant	Malignant
WH298	Benign	0.0047	Weihai	Benign	Benign	Benign	Benign
WH299	Benign	0.0011	Weihai	Benign	Benign	Benign	Benign
WH300	Malignant	0.4685	Weihai	Malignant	Malignant	Malignant	Malignant

Table S1 Continued

Patient ID	Pathological examination result	Probability predicted to be malignant by THCaDxNLP	Test set	Radiologist1 interpretation without THCaDxNLP	Radiologist2 interpretation without THCaDxNLP	Radiologist3 interpretation with THCaDxNLP	Radiologist4 interpretation with THCaDxNLP
WH301	Benign	0.0935	Weihai	Benign	Benign	Benign	Benign
WH302	Benign	0.0206	Weihai	Benign	Benign	Benign	Benign
WH303	Benign	0.0016	Weihai	Benign	Benign	Benign	Benign
WH304	Benign	0.0848	Weihai	Benign	Benign	Benign	Benign
WH305	Malignant	0.9941	Weihai	Malignant	Malignant	Malignant	Malignant
WH306	Benign	0.0125	Weihai	Benign	Benign	Benign	Benign
WH307	Benign	0.0009	Weihai	Benign	Benign	Benign	Benign
WH308	Benign	0.0286	Weihai	Benign	Benign	Benign	Benign
WH309	Malignant	0.9719	Weihai	Malignant	Malignant	Malignant	Malignant
WH310	Malignant	0.9891	Weihai	Malignant	Malignant	Malignant	Malignant
WH311	Malignant	0.9791	Weihai	Malignant	Malignant	Malignant	Malignant
WH312	Malignant	0.9752	Weihai	Malignant	Malignant	Malignant	Malignant
WH313	Benign	0.0115	Weihai	Benign	Benign	Benign	Benign
WH314	Malignant	0.9927	Weihai	Malignant	Benign	Malignant	Malignant
WH315	Benign	0.8052	Weihai	Benign	Benign	Benign	Benign
WH316	Malignant	0.0187	Weihai	Malignant	Malignant	Malignant	Malignant
WH317	Malignant	0.6878	Weihai	Malignant	Benign	Malignant	Malignant
WH318	Malignant	0.9759	Weihai	Malignant	Malignant	Malignant	Malignant
WH319	Benign	0.2810	Weihai	Benign	Benign	Benign	Benign
WH320	Malignant	0.9714	Weihai	Malignant	Benign	Malignant	Malignant
WH321	Malignant	0.3685	Weihai	Malignant	Benign	Malignant	Malignant
WH322	Malignant	0.6163	Weihai	Malignant	Benign	Malignant	Malignant
WH323	Malignant	0.9659	Weihai	Malignant	Malignant	Malignant	Malignant
WH324	Malignant	0.9140	Weihai	Malignant	Malignant	Malignant	Malignant
WH325	Malignant	0.9476	Weihai	Malignant	Benign	Malignant	Malignant
WH326	Malignant	0.0124	Weihai	Malignant	Benign	Benign	Malignant
WH327	Benign	0.0043	Weihai	Benign	Benign	Benign	Benign
WH328	Benign	0.0170	Weihai	Benign	Benign	Benign	Benign
WH329	Benign	0.0674	Weihai	Benign	Benign	Benign	Benign
WH330	Benign	0.1829	Weihai	Benign	Benign	Benign	Benign
WH331	Malignant	0.4712	Weihai	Benign	Benign	Malignant	Malignant
WH332	Malignant	0.9937	Weihai	Malignant	Benign	Malignant	Malignant
WH333	Benign	0.7072	Weihai	Benign	Benign	Benign	Benign

Table S1 Continued

Patient ID	Pathological examination result	Probability predicted to be malignant by THCaDxNLP	Test set	Radiologist1 interpretation without THCaDxNLP	Radiologist2 interpretation without THCaDxNLP	Radiologist3 interpretation with THCaDxNLP	Radiologist4 interpretation with THCaDxNLP
WH334	Malignant	0.9918	Weihai	Malignant	Malignant	Malignant	Malignant
WH335	Benign	0.0016	Weihai	Benign	Benign	Benign	Benign
WH336	Malignant	0.8346	Weihai	Malignant	Malignant	Malignant	Malignant
WH337	Benign	0.2003	Weihai	Benign	Benign	Benign	Benign
WH338	Benign	0.0011	Weihai	Benign	Benign	Benign	Benign
WH339	Malignant	0.9927	Weihai	Malignant	Malignant	Malignant	Malignant
WH340	Malignant	0.8769	Weihai	Malignant	Malignant	Malignant	Malignant
WH341	Malignant	0.9813	Weihai	Malignant	Malignant	Malignant	Malignant
WH342	Benign	0.0010	Weihai	Benign	Benign	Benign	Benign
WH343	Benign	0.3405	Weihai	Benign	Benign	Benign	Benign

Table S2 Classification performance of THCaDxNLP across five test sets

	TMUCIH test set (n = 439)	TGH test set (n = 186)	TFCH test set (n = 82)	Weihai test set (n = 343)	Chengde test set (n = 171)
Accuracy (95% CI)	0.866 (0.830–0.896)	0.801 (0.736–0.856)	0.915 (0.832–0.965)	0.875 (0.835–0.908)	0.854 (0.792–0.903)
Sensitivity (95% CI)	0.926 (0.889–0.954)	0.735 (0.636–0.819)	0.867 (0.693–0.962)	0.886 (0.828–0.930)	0.918 (0.838–0.966)
Specificity (95% CI)	0.755 (0.679–0.820)	0.875 (0.787–0.936)	0.942 (0.841–0.988)	0.864 (0.804–0.911)	0.791 (0.690–0.871)
Positive predictive value (95% CI)	0.874 (0.831–0.909)	0.867 (0.775–0.932)	0.897 (0.726–0.978)	0.860 (0.800–0.909)	0.812 (0.720–0.885)
Negative predictive value (95% CI)	0.848 (0.777–0.903)	0.748 (0.652–0.828)	0.925 (0.818–0.979)	0.889 (0.832–0.932)	0.907 (0.817–0.962)
Kappa[a]	0.698	0.604	0.815	0.749	0.708
F_1[b]	0.899	0.796	0.881	0.873	0.862

[a]Measures the agreement between predicted classification with pathological report. [b]Harmonic average of the precision and recall rate.
